# Thermal Synthesis of Carbamic Acid and Its Dimer in Interstellar Ices: A Reservoir of Interstellar Amino Acids

**DOI:** 10.1021/acscentsci.3c01108

**Published:** 2023-11-29

**Authors:** Joshua H. Marks, Jia Wang, Bing-Jian Sun, Mason McAnally, Andrew M. Turner, Agnes H.-H. Chang, Ralf I. Kaiser

**Affiliations:** † W. M. Keck Research Laboratory in Astrochemistry, University of Hawai’i at Manoa, Honolulu, Hawaii 96822, United States; ‡ Department of Chemistry, University of Hawai’i at Manoa, Honolulu, Hawaii 96822, United States; § Department of Chemistry, National Dong Hwa University, Hualien 974, Taiwan

## Abstract

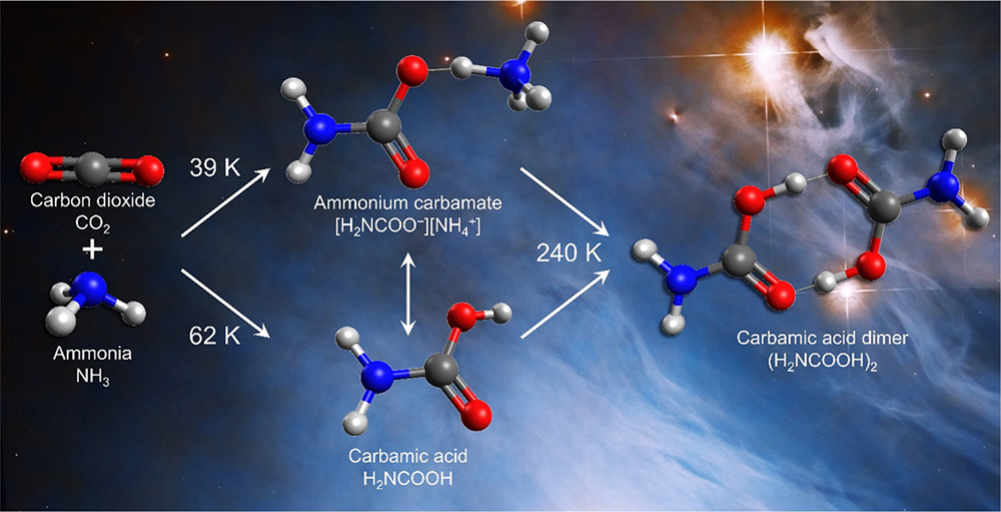

Reactions in interstellar ices are shown to be capable of producing key prebiotic molecules without energetic radiation that are necessary for the origins of life. When present in interstellar ices, carbamic acid (H_2_NCOOH) can serve as a condensed-phase source of the molecular building blocks for more complex proteinogenic amino acids. Here, Fourier transform infrared spectroscopy during heating of analogue interstellar ices composed of carbon dioxide and ammonia identifies the lower limit for thermal synthesis to be 62 ± 3 K for carbamic acid and 39 ± 4 K for its salt ammonium carbamate ([H_2_NCOO^–^][NH_4_^+^]). While solvation increases the rates of formation and decomposition of carbamic acid in ice, the absence of solvent effects after sublimation results in a significant barrier to dissociation and a stable gas-phase molecule. Photoionization reflectron time-of-flight mass spectrometry permits an unprecedented degree of sensitivity toward gaseous carbamic acid and demonstrates sublimation of carbamic acid from decomposition of ammonium carbamate and again at higher temperatures from carbamic acid dimers. Since the dimer is observed at temperatures up to 290 K, similar to the environment of a protoplanetary disk, this dimer is a promising reservoir of amino acids during the formation of stars and planets.

## Abstract 2

The carbamic acid dimer forms and remains in model interstellar ice at temperatures up to 290 K. Near young stellar objects it would provide a feedstock for amino acids needed for the origins of life.

## Introduction

**Figure 1:**
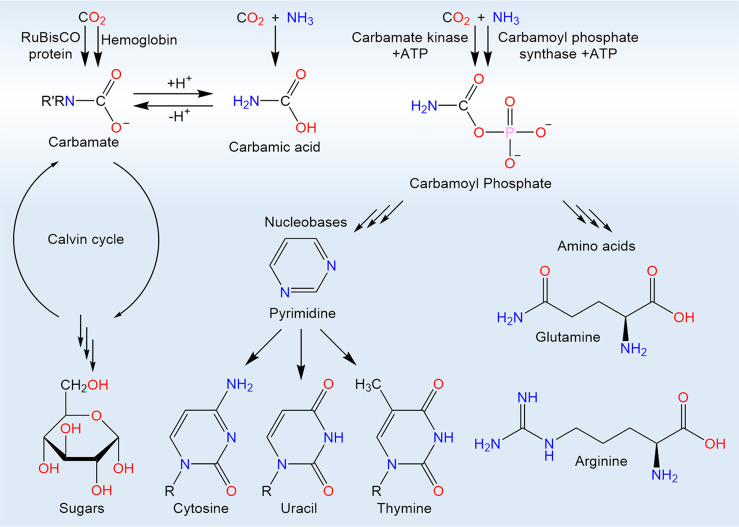
Additions of ammonia and amines to carbon dioxide are fundamental reactions in contemporary biochemical pathways. Biological sugar synthesis is initiated by carbon dioxide capture with amines, as is carbon dioxide transport by hemoglobin. Formation of carbamoyl phosphate is the first step in the synthesis of pyrimidine-derived nucleobases and the amino acids glutamine and arginine.

The incorporation of nitrogen into biological molecules represents a vital process in all living organisms.^1^ The proteins carbamoyl phosphate synthase and carbamate kinase both produce carbamoyl phosphate (H_2_NCOOPO_3_^2-^), which initiates the fixation of ammonia (NH_3_) toward the synthesis of pyrimidine nucleobases (cytosine, thymine, and uracil) along with amino acids arginine and glutamine (Figure 1).^2−5^ Functionalized carbamates (RR′NCOOR″/RR′NCOO^–^) play key roles in carbon dioxide (CO_2_) transport by hemoglobin and in carbon dioxide capture by the RuBisCO protein for sugar synthesis in plants.^4,6−8^ The urethane group (RNHCOOR′), a specific class of substituted carbamates, is the fundamental unit of polyurethanes, a common class of synthetic polymers.^9^ Carbamic acid (H_2_NCOOH, **1**) might be considered the simplest amino acid, though the lack of an additional carbon between the carboxyl and amino groups gives it a different chemistry than proteinogenic amino acids (H_2_NCH(R)COOH). It is the conjugate acid to the carbamate ion (NH_2_COO^–^) and a prototype molecule in studying the formation and reactions of chemically complex carbamates in which the hydrogen atom of the amino group is replaced by an organic side chain.^10^ Therefore, in consideration of the importance of carbamates in contemporary biochemistry, elucidating the synthesis of carbamates and their reactions in astrophysical environments is crucial to expanding our fundamental knowledge on the abiotic origin of biorelevant molecules in the interstellar medium (ISM) and their role in the origins of life on Earth. Here, we document the abiotic synthesis of **1** in interstellar analogue ices and identify the temperature-dependent sequence of reactions along with the effects of both ionizing radiation and sublimation to the abundance of carbamic acid.

**Figure 2:**
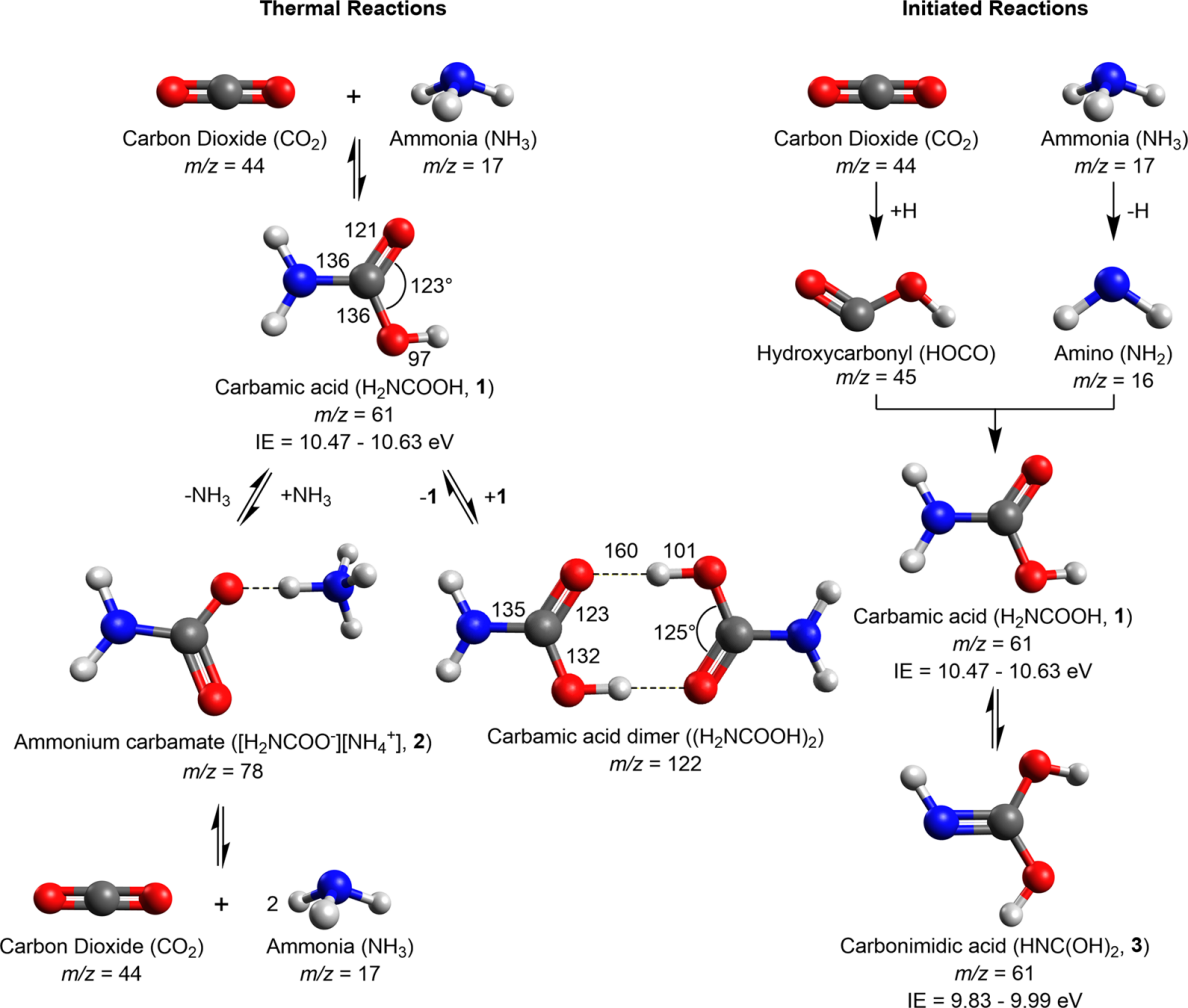
Reaction scheme for thermal and initiated reactions of carbon dioxide and ammonia in interstellar analog ices including mass-to-charge ratio (*m*/*z*) and ionization energy (IE). Carbamic acid (H_2_NCOOH, **1**) can be formed by addition of ammonia to carbon dioxide or by radical-radical recombination. Ammonium carbamate ([H_2_NCOO^–^][NH_4_^+^], **2**) can be formed by acid-base reactions of **1** and ammonia or by termolecular reactions of two molecules of ammonia with carbon dioxide. Amide-iminol tautomerization of **1** may result in carbonimidic acid (HNC(OH)_2_, **3**) with initiation. Calculated structural parameters are in units of pm for bond lengths and degrees for bond angles.

The synthesis of **1** in interstellar analog ices has been observed through both thermal reactions and nonequilibrium processes initiated by energetic particles, such as low-energy electrons, ultraviolet (UV) radiation, and galactic cosmic ray (GCR) proxies in ices containing ammonia and carbon dioxide with other known interstellar ice components such as water (H_2_O), carbon monoxide (CO), and methane (CH_4_).^11−24^ However, in these ices formation pathways to **1** have proven elusive (Figure 2); a concerted reaction toward ammonium carbamate ([H_2_NCOO^–^][NH_4_^+^], **2**) via termolecular reactions of carbon dioxide and two ammonia molecules has been hypothesized to produce **2**, which decomposes back into acid (**1**) and base (ammonia) components at “elevated temperatures” greater than 240 K.^12,18,25^ Nucleophilic reactions of **1** have been implicated in the preparation of complex organic molecules (COMs) — organic molecules containing six or more atoms by astronomical definition — including hexamethylenetetramine (*c*-N_4_(CH_2_)_6_)^13^ and glycine (H_2_NCH_2_COOH)^11^ in astrophysical environments, but confirmation of this pathway has been elusive. Computational analysis of the reactive ammonia–carbon dioxide system has proven to be an enduring but fruitful challenge. Molecule **1** is a predicted precursor to isocyanic acid (HNCO)^26,27^ and the biomolecule urea (H_2_NCONH_2_),^27−29^ both of which have been observed in the ISM toward the Sagittarius B2 giant molecular cloud (Sgr B2).^24,27,30−35^ The barrier to the bimolecular gas-phase addition of ammonia to carbon dioxide was calculated to be 199 kJ mol^–1^. In the gas phase, **1** is 51 kJ mol^–1^ higher in energy than ammonia and carbon dioxide and is inherently unstable; however, the 149 kJ mol^–1^ barrier to dissociation represents an unattainable amount of internal energy at the temperatures of dense interstellar molecular clouds (10 K) and star-forming regions up to 300 K.^34,36,37^ Solvation by protic solvents, e.g., water and ammonia, in a condensed environment such as ices reduces the barrier to formation/dissociation by solvent-assisted proton transfer. Furthermore, thermodynamically favorable polar solvation or dimerization stabilizes **1** below the energy of its reactants.^18,24,34^ Experiments that produced **1** and **2** in analogs of interstellar ices revealed that their formation proceeds at temperatures as low as 80 K, providing evidence that the solvation offered by interstellar ices may both facilitate the reaction and stabilize the reaction products, leading to highly efficient production of prebiotic molecules with nothing more than the thermal energy available in the star-forming regions of molecular clouds or circumstellar environments.^17−20,24,38^

Prior efforts to study the chemical dynamics of this system in ices have relied on Fourier transform infrared spectroscopy (FTIR) and quadrupole mass spectrometry (QMS); however, the instability of **1** when subjected to hard (dissociative) ionization from electron impact and the absence of gaseous **2**, a salt that decomposes into ammonia and carbon dioxide on heating, have hindered the gas-phase detection.^17,18,20−22,39−46^ The high sublimation/dissociation temperature of these species implicates them as critical reservoirs of amino and carboxylic acid/carboxylate moieties within interstellar ices condensed onto nanoparticles at temperatures far in excess of the sublimation temperatures of ammonia (105 K) or carbon dioxide (110 K).^18,22^ The presence of these molecules in space is still unproven, but the James Webb space telescope (JWST)^47^ is able to detect these species if they are present in ices with infrared spectroscopy while millimeter/submillimeter telescopes such as the Atacama large millimeter array (ALMA)^48^ can probe regions with sufficient temperature for desorption. The efficient formation of **1** and **2** and their abundance in protostellar environments and protoplanetary disks during star formation would lead to significant chemical processing in ices by the intense UV and vacuum UV radiance of protostars.^49^ Processing and radical reactions in ices can result in substituted carbamates which are thermally stable and serve as precursors to key biochemical molecules.^50^ Eventual delivery of these molecules to early Earth or newly formed exoplanets by comets and meteorites provides a facile means of introducing carbamates and their derivatives during the geologic epoch during which life originated.^51−54^

Here, we present an investigation of the formation and interconversion pathways of **1** and **2** along with the dimerization of **1** in binary ammonia–carbon dioxide interstellar analog ices utilizing temperature-resolved condensed-phase FTIR spectroscopy combined with gas-phase detection via isomer-selective single-photon photoionization time-of-flight mass spectrometry (PI-ReToF-MS).^55^ This simultaneous condensed- and gas-phase analysis scheme permits unparalleled insights into the competing processes of decomposition, dimerization, and sublimation. The high sensitivity and soft (fragmentation-free) nature of photoionization is exploited to produce compelling evidence of the stability of carbamic acid along with its dimer in the gas phase and demonstrating that this molecule is a key molecular reservoir of ammonia in astrophysical ices at temperatures up to 290 K — well above the sublimation temperature of ammonia at 105 K under ultrahigh vacuum and interstellar conditions.^49,56^

## Results

### Fourier Transform Infrared Spectroscopy (FTIR)

**Figure 3:**
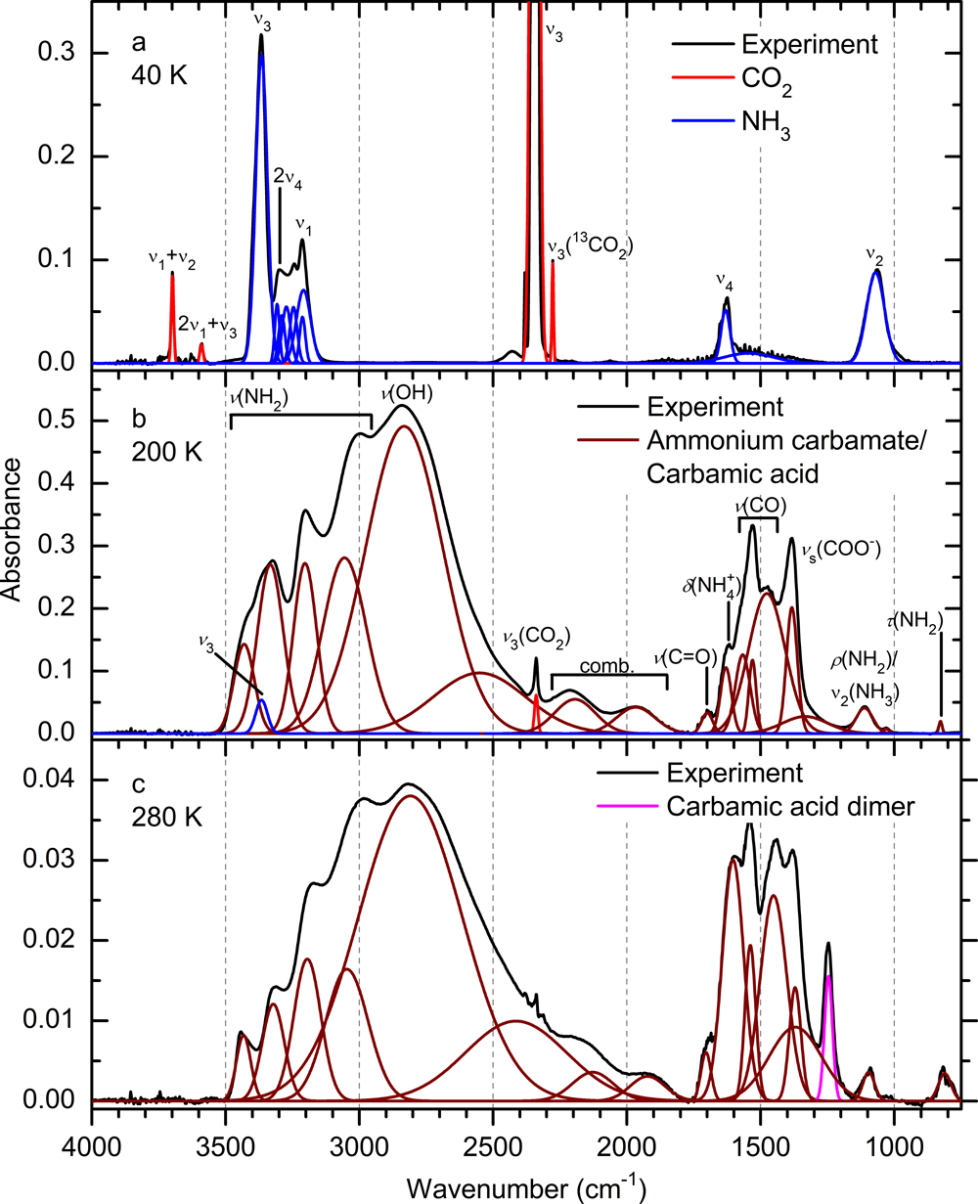
Fourier transform infrared spectra (FTIR) of ammonia–carbon dioxide ice at various temperatures. The spectrum at (a) 40 K shows only peaks assignable to carbon dioxide (red) and ammonia (blue). At (b) 200 K peaks assigned to ammonium carbamate (**2**) and carbamic acid (**1**) are observed in dark red. At (c) 280 K the peaks assigned to carbamic acid are more pronounced and a peak assigned here to its dimer (magenta) is observed in the C–O stretching region. Labels indicate stretching (ν), bending (δ), rocking (ρ), and twisting (τ).

Ammonia–carbon dioxide ices were deposited from the gas phase onto a silver substrate maintained at 5–10 K and examined with FTIR spectroscopy to determine the relative abundance of both components (Figure 3a, Figures S1–S5, Table S1). Spectra show infrared absorptions that can be attributed exclusively to carbon dioxide and ammonia, demonstrating that no detectable reactions occur during deposition. Infrared spectra were also recorded continuously during the temperature-programmed desorption (TPD) phase of the experiment in which ice was heated at a rate of 1 K minute^–1^. Compared to the freshly deposited ice, spectra at 200 K show new infrared absorptions (Figure 3b); this is attributed to the thermal formation and reactions toward carbamic acid (**1**) and ammonium carbamate (**2**), which are more infrared active than either of its reactants. Even though carbon dioxide and ammonia typically sublime at 100–110 K,^57,58^ their continuing presence within the ice at 200 K is evidenced by their absorptions at 2339 cm^–1^ (ν_3_, CO_2_) and 3366 cm^–1^ (ν_3_, NH_3_). New absorptions include NH stretching (3400–3200 cm^–1^), while the presence of the broad absorption from 3200–2000 cm^–1^ is a clear indication of hydroxyl (−OH) groups engaging in hydrogen bonding in a polar environment. Since **1** and **2** share most of the same moieties, their vibrational spectra are similar. However, prior spectroscopic investigations of these species permit the assignment of unique vibrations to each species. The carbonyl (C=O) moiety is found only in **1**, observed at 1698 cm^–1^.^12^ The detection of **2** is accomplished by its carbonate (COO^–^) stretch at 1383 cm^–1^ and NH bending of the ammonium (NH_4_^+^) ion at 1629 cm^–1^.^12^ Solid ices composed of these species have been observed to decompose back into their component gaseous reactants at 240–250 K.^14,18,24,39,41^ However, high-sensitivity FTIR and a slow heating rate during TPD reveal 7% of the ice composed of **1** persisting to temperatures of 280 K.

The 280 K infrared spectrum shows the presence of key functional groups observed at 200 K, though the maximum absorbance has decreased by an order of magnitude; the peak positions of multiple absorptions are perturbed (Figure 3c). Furthermore, a new vibration was observed at 1247 cm^–1^, which can be assigned to the dimer of **1**, with intermolecular bonding between the dipoles of the carboxylic acids. The vibration appears in the range 1320–1210 cm^–1^ where the C–O stretch of carboxylic acid dimers is expected.^59^ If either the carbon or oxygen participating in a C–O stretch is substituted with a heavier isotope, the corresponding vibrational band should exhibit a red shift. FTIR spectra were collected for ammonia-^15^N–carbon dioxide and ammonia–carbon dioxide-^18^O ices (Figure S6). The ^15^N-labeled ice demonstrates a negligible shift in the position of this vibration (1249 cm^–1^), showing that the vibration in question does not include a significant motion of nitrogen. In contrast, the ^18^O-labeled ice exhibits a 19 cm^–1^ red shift to 1228 cm^–1^, indicating the significant involvement of the oxygen in the vibrational motion (Table S2). Computational quantum chemical analysis (unscaled, Tables S3–S6) predicts a vibrational motion that is a mixture of both C–O stretching with COH bending contributions at 1342 cm^–1^ and red shifts 11 cm^–1^ to 1331 cm^–1^, confirming an infrared absorption that red-shifts in response to ^18^O isotopic labeling in this region which is present in the dimer but not the monomer.^60^ These calculations make use of the gas-phase B3LYP/cc-pVTZ methodology, and the predicted wavelength without isotopic labeling is 95 cm^–1^ higher than the observed position. This is likely the result of a combination of anharmonicity and comparison between gas-phase calculations and condensed phase measurements; the position of this vibrational band is within the range in which other carboxylic acid dimers have been found to vibrate.^59^

**Figure 4:**
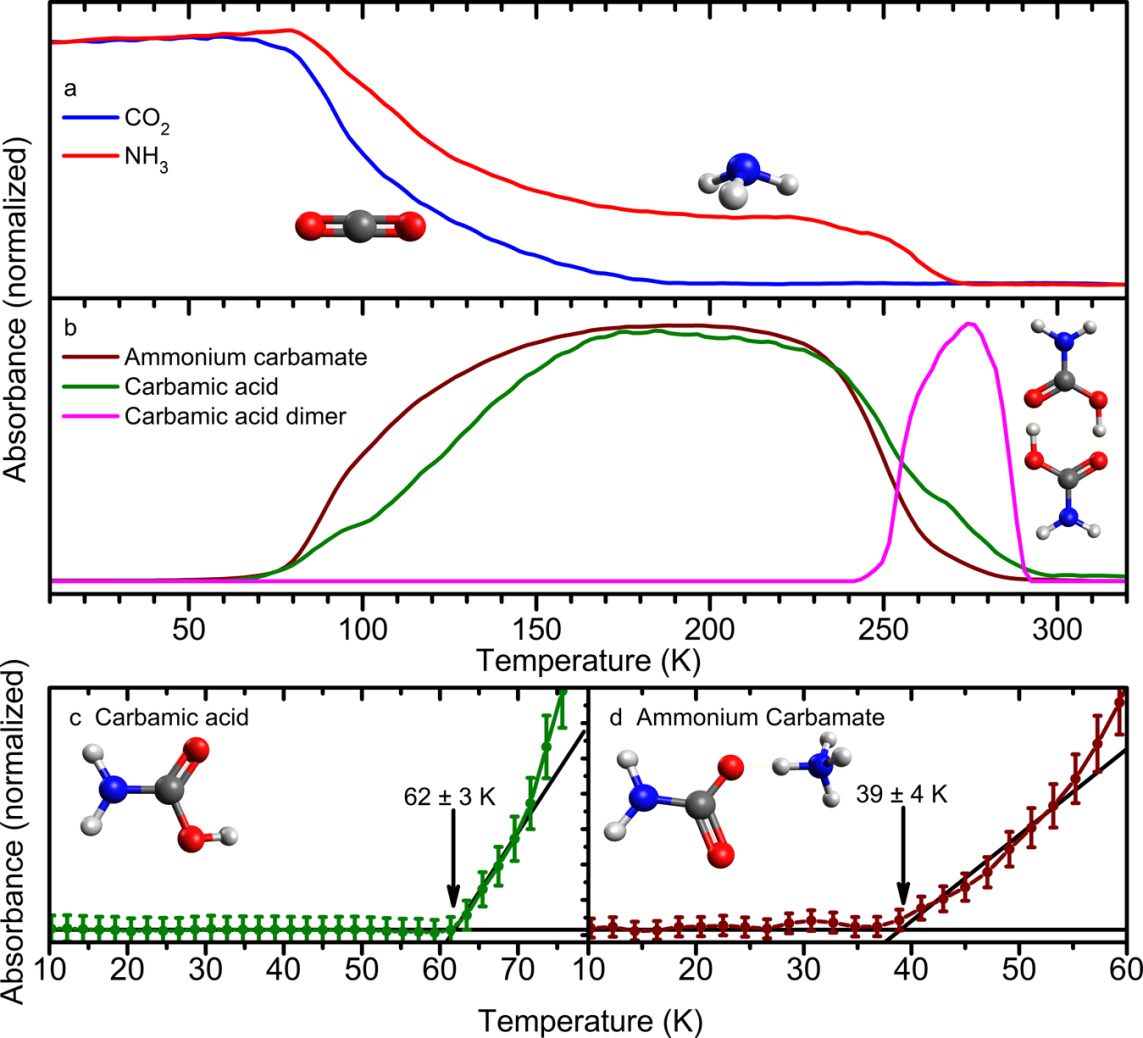
Normalized FTIR intensity of reactants and products. Reactants (a) CO_2_ (blue, ν_1_+ν_3_) and NH_3_ (red, ν_1_+ν_4_) begin to deplete at a significant rate at 80 K. (b) Carbamic acid (**1**, ν(C=O), green) and ammonium carbamate (**2**, ν_as_(COO^–^), dark red) form at an increased rate at 80 K and begin to decompose at 240 K; error is comparable to line thickness for panels a and b. Carbamic acid dimer is found to begin to form during decomposition of ammonium carbamate but leaves the condensed phase at 290 K. Low-temperature formation is interpolated to commence for (c) carbamic acid at 62 ± 3 K and (d) ammonium carbamate at 39 ± 4 K.

The data presented in Figure 4 are temperature-dependent absorbance measurements of bands uniquely assigned to molecules within the ice. The resulting FTIR-TPD profiles are normalized to the same arbitrary maximum absorbance such that the relative rates of formation and the temperature dependence thereof are apparent. The experimentally observed temperature-resolved infrared absorbance of carbon dioxide (ν_1_+ν_3_, 3620 cm^–1^) and ammonia (ν_1_+ν_4_, 5007 cm^–1^) show that while carbon dioxide has nearly entirely sublimed or reacted by the time TPD reached 200 K, unreacted ammonia is present in the ice until 270 K (Figure 4a). At 80 K the abundances of both reactant species begin to drop rapidly as they are consumed by reactions. Because there is no sudden loss of these molecules apparent at 100–110 K, where they should sublime, it is likely that chemical reactions are the primary loss mechanism. Infrared absorption intensities assigned to **1** (C=O stretching, 1698 cm^–1^) and **2** (COO^–^ asymmetric stretching, 1383 cm^–1^) show a significant increase in their respective rates of formation at 80–100 K, corresponding to the decrease in the absorption from carbon dioxide and ammonia. At temperatures below 120 K the formation rate of **2** exceeds that of **1**, and at 250 K the signal from **2** is found to rapidly diminish in comparison to **1**. The depletion of **2** at 250 K is due to its decomposition through routes which result in either **1** and ammonia, or carbon dioxide and two molecules of ammonia. The signal assigned to the dimer of **1** (C–O stretch, 1247 cm^–1^) occurs during the greatest rate of loss of **2** with an onset at 240 K and a rapid increase in absorption at 250 K, and culminates in its rapid decomposition into gaseous products at 290 K. The dimer has been cited as being responsible for changes in the infrared spectrum between 240 and 140 K.^17^ However, the dipole moment of **1** should be more strongly attracted to the charges of **2** than the weaker charge density represented by a dipole. The formation of dimers of **1** while **2** is still abundant is unlikely, and the previously observed changes in infrared absorption is readily attributed to changes in the temperature-dependent equilibrium concentrations of **1** and **2**.

### Photoionization Reflectron Time-of-Flight Mass Spectrometry (PI-ReToF-MS)

**Figure 5:**
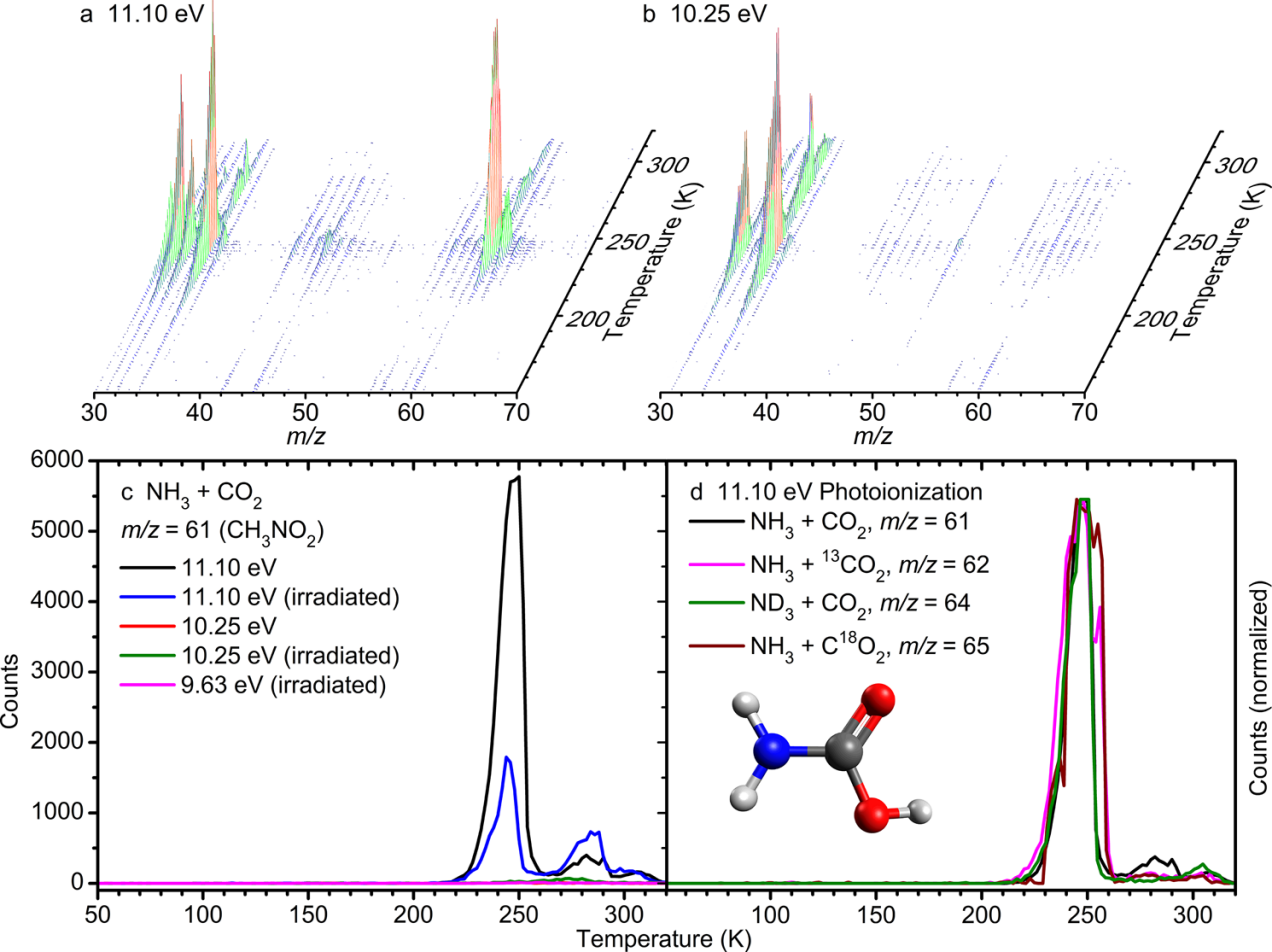
Photoionization reflectron time-of-flight mass spectrometry (PI-ReToF-MS) data as a function of temperature during temperature-programmed desorption (TPD) for carbon dioxide–ammonia ices with (a) 11.10 eV and (b) 10.25 eV photoionization. (c) Ionization is observed at *m*/*z* = 61 at 11.10 eV but not at 10.25 eV with peaks at 250 and 280 K, while irradiation is found to reduce the quantity of carbamic acid (**1**) detected in the gas phase by 70%. (d) The same peak at 250 K is observed at the appropriate *m*/*z* with isotopic labeling of each atom.

The PI-ReToF-MS technique was used to provide isomer-selective mass analysis of molecules subliming during TPD. With the use of photoionization, a species can only be detected if the photon energy is in excess of adiabatic ionization energy.^61−64^ Since molecules are detected in the gas phase, these high-sensitivity measurements provide information on both the sublimation of **1** and the decomposition of **2** to yield **1**. Mass spectra obtained with the PI-ReToF technique are plotted as a function of temperature in Figure 5a,b. These mass spectra demonstrate that several molecules present within the ice are detected during the sublimation or decomposition of **1** and **2** at 250 K, though the signal at *m*/*z* = 61 is the most intense. The coincidence of peaks at 250 K is unlikely to be due to identical kinetics governing sublimation for multiple species present within the ice, but rather cosublimation of most of the ice as the bulk of it, i.e., **1** and **2**, is converted to gas through sublimation and decomposition.

The adiabatic ionization energy of **1** is calculated to be 10.47–10.63 eV; its elusive amide-iminol tautomer, carbonimidic acid (HNC(OH)_2_, **3**), is predicted to have an ionization energy of 9.83–9.99 eV.^60^ With photoionization at 11.10 eV both **1** and **3** can be detected if present, but at 10.25 eV only **3** can be detected (Table S7). Ices were irradiated with energetic electrons as proxies of GCRs penetrating deep inside cold molecular clouds (Table S8); these studies were carried out to determine if energetic processing can also result in the formation of **3**, the effects of irradiation on the condensed phase and a comparison to thermal processing having been the subject of prior work.^40,41^ During TPD of the unirradiated ice, photoionization with 11.10 eV photons *m*/*z* = 61 was detected above 210 K and peaks three separate times as the temperature approached 320 K, while irradiation of the ice with a dose of 1.24 ± 0.03 eV molecule^–1^ of ammonia and 3.2 ± 0.1 eV molecule^–1^ of carbon dioxide reduces the peak number of counts from 5,780 to 1,790 (Figure 5c). When the photon energy is reduced to 10.25 eV a signal is detected for *m*/*z* = 61 peaking at 276 K in irradiated ices, but when photon energy is reduced to 9.63 eV this signal is still present and therefore cannot be assigned to **3**. The molecular formula of the ion observed at 11.10 eV was determined through the use of isotopic labeling (Figure 5d). The signal observed at 11.10 eV was detected during TPD of ammonia–carbon dioxide-^13^C ice at *m*/*z* = 62, ammonia-*d*_3_–carbon dioxide ice at *m*/*z* = 64, and ammonia–carbon dioxide-^18^O_2_ ice at *m*/*z* = 65. These results show that the molecular formula of the ion detected at 11.10 eV and *m*/*z* = 61 in ammonia and carbon dioxide ice must be CO_2_NH_3_ with an ionization energy between 11.10 and 10.25 eV, confirming the assignment of **1** to this signal.

## Discussion

The combination of condensed-phase (FTIR) and high-sensitivity gas-phase (PI-ReToF-MS) measurements employed here permits unprecedented insights into the physical and chemical processes occurring in ammonia–carbon dioxide ices. The sequence of both chemical reactions and physical changes in the ice during TPD can be determined only through the information obtained in both phases. The first event occurs at 39 ± 4 K (Figure 4d), when **2** is initially identified via its infrared spectrum. There are two pathways through which this synthesis can proceed, nucleophilic addition of ammonia to carbon dioxide [1] followed by a Brønsted-Lowry acid-base reaction to form a salt [2] or through a concerted reaction [3].
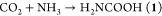
1
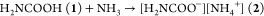
2
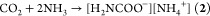
3Reaction [2] requires the presence of **1**; however, it is not observed until the temperature has increased to 62 ± 3 K. Therefore, synthesis of **2** must initially proceed through the concerted process demonstrated by reaction [3].

Top-down or dissociative reactions [1a] to [3a], the reverse of reactions [1] to [3], play roles in both the synthesis of **1** and the ultimate decomposition into carbon dioxide and ammonia.
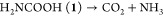
1a
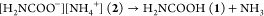
2a
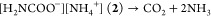
3a

Formation of **1** may proceed through either a bottom-up process represented by [1] or can be the result of dissociation [2a] of **2** into its component acid and base. It may be the case that both reactions [1] and [2a] are responsible for the presence of **1** in this ice. The depletion of carbon dioxide within the ice during TPD at 190 K (Figure 4a) would inhibit reaction [1]; however, at higher temperatures, **1** and **2** should exist in a temperature-dependent equilibrium. Furthermore, the relative abundances of these species are found to vary during their formation and depletion from the ice. Changes in their relative abundances within the ice are measured by the changes in the intensities of the molecule-specific vibrational absorptions (Figure 4). The normalized infrared absorption of **2** is in excess of that of **1** during their accumulation in the ice at temperatures lower than 174 K. This may represent a period during which reaction [1] is dominant. At 236 K the normalized intensity of **1** begins to exceed that of **2** (Figure 4b); at this temperature reaction [2a] is a plausible mechanism by which the relative abundances may change. It is also possible that reaction [3a] is responsible for the depletion of **2** at higher temperatures. Similar catalysis of reaction [1a] may be responsible for the reduction in the amount of gas-phase **1**. Irradiation of ammonia–carbon dioxide ice results in a substantial decrease in the amount of **1** detected in the gas phase. Chemical reactions accessible only through initiation by electron irradiation simulating the effects of GCR produce significant amounts of molecules that must interfere with the formation of **1** and **2**, or potentially accelerate the rate of decomposition, ultimately resulting in a lower abundance of **1** in the gas phase. The initiated reaction [4] that produces **3** may not occur due to the effect of the polar ice in which these experiments were conducted.
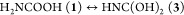
4

The similar isomerization of acetic acid (CH_3_COOH) to 1,1-ethenediol (CH_2_C(OH)_2_), which are isoelectronic with **1** and **3** respectively, was not previously found to occur in polar ices, while bottom-up synthesis of 1,1-ethenediol has been demonstrated only in apolar ices.^65^

At higher temperatures, the gas-phase detection of **1** provides key information about the processes occurring within the ice. During the PI-ReToF-MS measurement (Figure 5), the greatest abundance of **1** in the gas-phase is found at 250 K and corresponds to the largest rate of decrease in the FTIR signal for **2**. This is conclusive evidence that reaction [2a] occurs and results in the formation of gas-phase **1**, and whether reaction [2a] is responsible for the production of solid **1** cannot be confirmed. The second peak detected with PI-ReToF-MS is found at 285 K, and it corresponds to the rapid decrease in the infrared absorption assigned to the dimer of **1** (Figure 4b). This feature has previously been identified in ices containing nearly equal amounts of carbon dioxide and ammonia, though its cause has remained uninvestigated.^39^ The dimer of **1** is the result of hydrogen bonding between carboxylic acid moieties dominated by dipole–dipole interactions. While **2** remains present in the ice, the charge-dipole interactions between **1** and **2** should predominate and disrupt the dimerization. The depletion of **2** and the onset of absorption attributed to the dimer occur simultaneously, supporting the concept that the presence of one molecule prevents the formation of the other. The fraction of **1** that ultimately forms dimers is relatively small, as demonstrated by the decrease in peak infrared absorption intensity. The NH stretching region should not be strongly affected by dimerization and demonstrates that the peak absorption intensity is reduced by 90% as temperature increases from 200 to 280 K (Figure 4). The dimerization of **1** results in an intermolecular bond with a dissociation energy of 75 kJ mol^–1^; no signal was detected for the dimer at *m*/*z* = 122 at 285 K in the gas phase, so dissociation of this intermolecular bond must be accomplished before or during sublimation.^24^ The highest temperature peak observed in the PI-ReToF mass spectra is found at 306 K and is the least intense. While it is possible there is still some component of the ice remaining on the wafer at this temperature, no infrared absorption is detectable, and without correlation with mass spectrometry, no cause of this peak is apparent.

As a result of the hydrogen bonding that forms the dimer of **1**, the C–O bond length decreases from 136 to 132 pm, while the C=O bond length increases from 121 to 123 pm. This is a result of each OH group accepting electron density from the C=O group of the dimeric partner, while sharing of the proton between the two oxygens incorporates some C–OH character into the C=O bonds, while some C=O character is incorporated into the C–OH bond. The resulting structure is a compromise between the carbon–oxygen single and double bonds. The structure of the dimer of **1** is similar to that of the dimer of acetic acid ((H_3_CCOOH)_2_), a prototype carboxylic acid dimer.^66,67^ Bond lengths for those bonds present in both dimers, i.e., C–O, C=O, and O–H, agree within 1 pm, while the OCO bond angles differ by less than 1°. The same degree of agreement is reached between calculated parameters of the monomer **1** and acetic acid for the parameters of the COOH group found in both molecules.^60,67^ This shows that the chemical bonding that governs the formation of these dimers is the same in both examples, and the substitution of the methyl (−CH_3_) group of acetic acid for the amino (−NH_2_) group has little effect on the dimer bond.

## Conclusion

Here, we report the thermal limits for the formation of carbamic acid (H_2_NCOOH, **1**) and ammonium carbamate ([H_2_NCOO^–^][NH_4_^+^] **2**) to be 62 ± 3 and 39 ± 4 K, respectively, in interstellar analog ices. Decomposition of **2** into ammonia (NH_3_), carbon dioxide (CO_2_), and **1** is found to be rapid at 250 K in a process that results in a significant proportion of **1** entering the gas phase. Despite the relative ease with which **1** can decompose in the condensed phases (ices), in isolation this molecule is predicted to face a 148 kJ mol^–1^ barrier to dissociation and should remain intact at the temperatures of dense molecular clouds.^34^ Therefore, experimental verification of the facile decomposition of **1** and its survival until gas-phase detection imply the critical role of solvation effects in its decomposition. Carbon dioxide and ammonia are prevalent in interstellar ices which contain up to 50% and 31% relative to water, respectively.^68,69^ The ease of formation presented by **1** by purely thermal reactions and the abundance of the reactants make this a prime candidate for interstellar detection; the laboratory measurement of its rotational or vibrational spectra is necessary to aid in detecting this molecule in space. After these laboratory measurements have been made, telescopes like the James Webb space telescope (JWST)^47^ or the Atacama large millimeter array (ALMA)^48^ can search for **1** (gaseous/solid) or **2** (solid) in star-forming regions where temperatures are ideal for their production and sublimation.

The fraction of **1** that remains in the studied ices above 250 K is found as a dimer binding through the carboxylic acid moieties, identified through a strongly red-shifted infrared absorption of the C–O stretch at 1247 cm^–1^. The stability of the dimer, 75 kJ mol^–1^ more favorable than the monomer, allows **1** to remain in the condensed phase, and available for reactions within interstellar and circumstellar ices, at temperatures up to 290 K.^24^ The low temperature at which **1** and **2** are formed implies that this process can proceed in ices found in dense molecular clouds at the earliest stages of star formation where temperatures reach up to 50 K.^36,37^ Ices observed spectroscopically in molecular clouds are predominantly water, unlike model ices employed here; however, the reaction products observed have also been identified with FTIR in model ices composed primarily of water.^14^ As gravitational collapse of these clouds drives star formation, the energetic environment produced in the vicinity of a protostar, known as a hot molecular core, reaches temperatures of 100–300 K, which are sufficient to drive the thermal synthesis of these molecules.^36,37^ The sublimation of ammonia and carbon dioxide proceeds at temperatures as low as 105 K when unperturbed, but the formation of **1** and/or **2** can trap these in the condensed phase. The ultraviolet emission of young stellar objects can drive radical reactions in ices by causing dissociation followed by radical–radical recombination.^70^ The presence of the amino (−NH_2_), ammonium (NH_4_^+^), carboxylic acid (−COOH), and carboxylate (−COO^–^) moieties present in **1** and **2** can contribute to the formation of more complex molecules containing these groups such as amino acids.^11,71−74^ Exchange of hydrogen for alkyl groups through this mechanism can produce substituted carbamates, which, unlike **1**, do not readily dissociate into gases. Delivery of these stable reaction products to newly formed terrestrial environments via meteorites and comets constitutes a plausible source of prebiotic molecules on the early Earth.^52−54^

## Supporting Information Available

Experimental and computational details and methods including FTIR spectra of freshly deposited ices, isotopically labeled ices at 280 K, and computed structures, energetics, and vibrational frequencies (PDF)
Transparent Peer Review report available (PDF)
