# Supplementary material for: Thermal Synthesis of Carbamic Acid and Its Dimer in Interstellar Ices: A Reservoir of Interstellar Amino Acids
Source: ACS Cent Sci. 2023 Nov 29;9(12):2241–50. doi: 10.1021/acscentsci.3c01108 (PMC10755733; doi:10.1021/acscentsci.3c01108)
Supplement: Supplementary file 1 — oc3c01108_si_001.pdf [file oc3c01108_si_001.pdf]

Supporting Information:

Thermal Synthesis of Carbamic Acid and its Dimer in Interstellar Ices: A Reservoir of Interstellar Amino Acids

Joshua H. Marks,[1,2] Jia Wang,[1,2] Bing-Jian Sun,[3] Mason McAnally, [1,2] Andrew M. Turner, [1,2] Agnes H.-H. Chang,[3*] Ralf I. Kaiser[1,2]*

[1] *W. M. Keck Research Laboratory in Astrochemistry, University of Hawai'i at Manoa, Honolulu, Hawaii 96822, USA*

[2] *Department of Chemistry, University of Hawai'i at Manoa, Honolulu, HI 96822, USA*

[3] *Department of Chemistry, National Dong Hwa University, Hualien 974, Taiwan*

* Corresponding Authors:

Agnes H.-H. Chang, hhchang@gms.ndhu.edu.tw

Ralf I. Kaiser, ralfk@hawaii.edu

Experimental Methods

The experiments described were carried out at the W. M. Keck Research Laboratory in Astrochemistry.[1-3] The apparatus is housed in a hydrocarbon-free stainless steel ultra-high vacuum (UHV) chamber which is maintained at a few 10^{-11} Torr by magnetically levitated turbomolecular pumps.[4] A closed cycle helium refrigerator (Sumitomo Heavy Industries, RDK-415E) cools a mirror-polished silver wafer (15.1×12.6 or 32.8×32.8 mm for PI-ReToF-MS or FTIR-TPD experiments, respectively) to 5–12 K. The refrigerator-wafer assembly is rotatable about the vertical axis because it is mounted on a doubly differentially pumped rotatable flange (Thermionics Vacuum Products, RNN-600/FA/MCO), and can be translated along its rotation axis via an adjustable bellows (McAllister, BLT106). Ices were prepared by passing ammonia (NH_3, Matheson, 99.99%; $^{15}NH_3$, Aldrich, 98% atom ^{15}N; ND_3, Aldrich, 99% atom D) and carbon dioxide (CO_2, Airgas, research grade; $^{13}CO_2$, Cambridge Isotope Laboratories, 99% ^{13}C, < 1% ^{18}O; $C^{18}O_2$, Aldrich, 95% atom ^{18}O) through separate glass capillary arrays (10 or 25.4 mm diameter for PI-ReToF-MS or FTIR-TPD experiments, respectively) directed at the cooled wafer. Partial pressures of each ice component were adjusted by the use of leak valves to achieve approximately a 1:1 ratio of $[NH_3]:[CO_2]$. Ice thickness was determined by monitoring the ice deposition with a helium-neon laser (CVI Melles-Griot, 25-LHP-230, 632.8 nm) at a 4° angle of incidence and measuring variations in reflected power due to thin film interference by the ice.[5] The index of refraction of the mixed ice is unknown though this parameter is necessary to determine ice thickness from interferometric measurements. This parameter was approximated by the average of the indexes of refraction of the two components, 1.33 for ammonia at 18 K and 1.27 ± 0.02 at 15 K for acetaldehyde, with an average of 1.32 ± 0.03.[6,7] Details of the composition and thickness of ices studied are reported in Table S1.

Fourier transform infrared (FTIR) spectra (Thermo Electron, Nicolet 6700) were measured in the range 6000–500 cm^{-1} with 230 spectra averaged over a two-minute period after ice deposition and used to calculate the relative abundance of the two components (Figures S1–S5). Relative concentrations of ammonia and carbon dioxide in ices were determined using integrated infrared absorption strength of the components. For ammonia, the integrated intensities of ν_2, ν_3, and ν_4 observed at 1077, 3394, and 1627 cm^{-1} have absorption coefficients of 2.1×10^{-17}, 2.3×10^{-17}, and 5.6×10^{-18} cm $molecule^{-1}$, respectively.[8] For carbon dioxide, the integrated intensities used for quantification were $\nu_1+\nu_3$ (3708 cm^{-1}, 1.8×10^{-18} cm $molecule^{-1}$), $2\nu_2+\nu_3$ (3600 cm^{-1}, $5.5 \times 10^{-}$

^{19} cm molecule^{-1}), and ν_3 ($^{13}CO_2$; 2283 cm^{-1}, 6.8 × 10^{-17} cm molecule^{-1}).[8] FTIR spectra were measured every 2 minutes during TPD.

The photoionization reflectron time-of-flight mass spectrometry (PI-ReToF-MS) technique has been discussed in detail elsewhere.[1] Ices were heated to 320 K with temperature programmed desorption (TPD) at a rate of 1 K min^{-1}. During TPD, pulsed 30 Hz coherent vacuum ultraviolet (VUV) light was passed 1–2 mm above the surface of the ice to photoionize subliming molecules. VUV light was produced via resonant difference four-wave mixing ($\omega_{VUV} = 2\omega_1 \pm \omega_2$) schemes (Table S7). After generation of the selected ω_1 and ω_2, the lasers were made collinear and directed through a lens (Thorlabs, LA5479, f = 300 mm) and focused into a jet of rare gas in the VUV generation vacuum chamber. Coherent VUV light exiting this chamber was separated from ω_1 and ω_2 by passing the collinear beams through an off-axis lithium fluoride (LiF) biconvex lens (Korth Kristalle, $R_1 = R_2 = 131.22$ mm) which imparts an angular separation between the three frequencies and directs only the VUV light through an aperture to the ionization region. Ions formed are mass-analyzed in a reflectron time-of-flight mass spectrometer (ReToF-MS; Jordan TOF Products) and detected with a dual microchannel plate (MCP) detector in the chevron configuration (Jordan TOF Products). MCP signal was amplified (Ortec, 9305) before discrimination and amplification to 4 V (Advanced Research Instruments Corp., F100-TD) and recorded by a multichannel scaler (FAST ComTec, MCS6A). Ion arrival times were recorded to 3.2 ns accuracy. New mass spectra were accumulated every two minutes during TPD.

Computational Methods

The geometries of carbamic acid dimer and its isotopologues and their cations were optimized by the hybrid density functional B3LYP[9-12] with the cc-pVTZ basis set and the harmonic frequencies obtained (Tables S3–S6). The corresponding coupled cluster[13-16] CCSD(T)/cc-pVDZ, CCSD(T)/cc-pVTZ, and CCSD(T)/cc-pVQZ energies are calculated and extrapolated to completed basis set limits,[17] CCSD(T)/CBS, with B3LYP/cc-pVTZ zero-point energy corrections. The adiabatic ionization energies are computed by taking the energy difference between the neutral and cationic species that correspond to similar conformation. The adiabatic ionization energies at this level of calculations (CCSD(T)/CBS//B3LYP/cc-pVTZ) are expected to be accurate within ± 0.05 eV.[18,19] The GAUSSIAN16 program[20] was utilized in the electronic structure calculations.

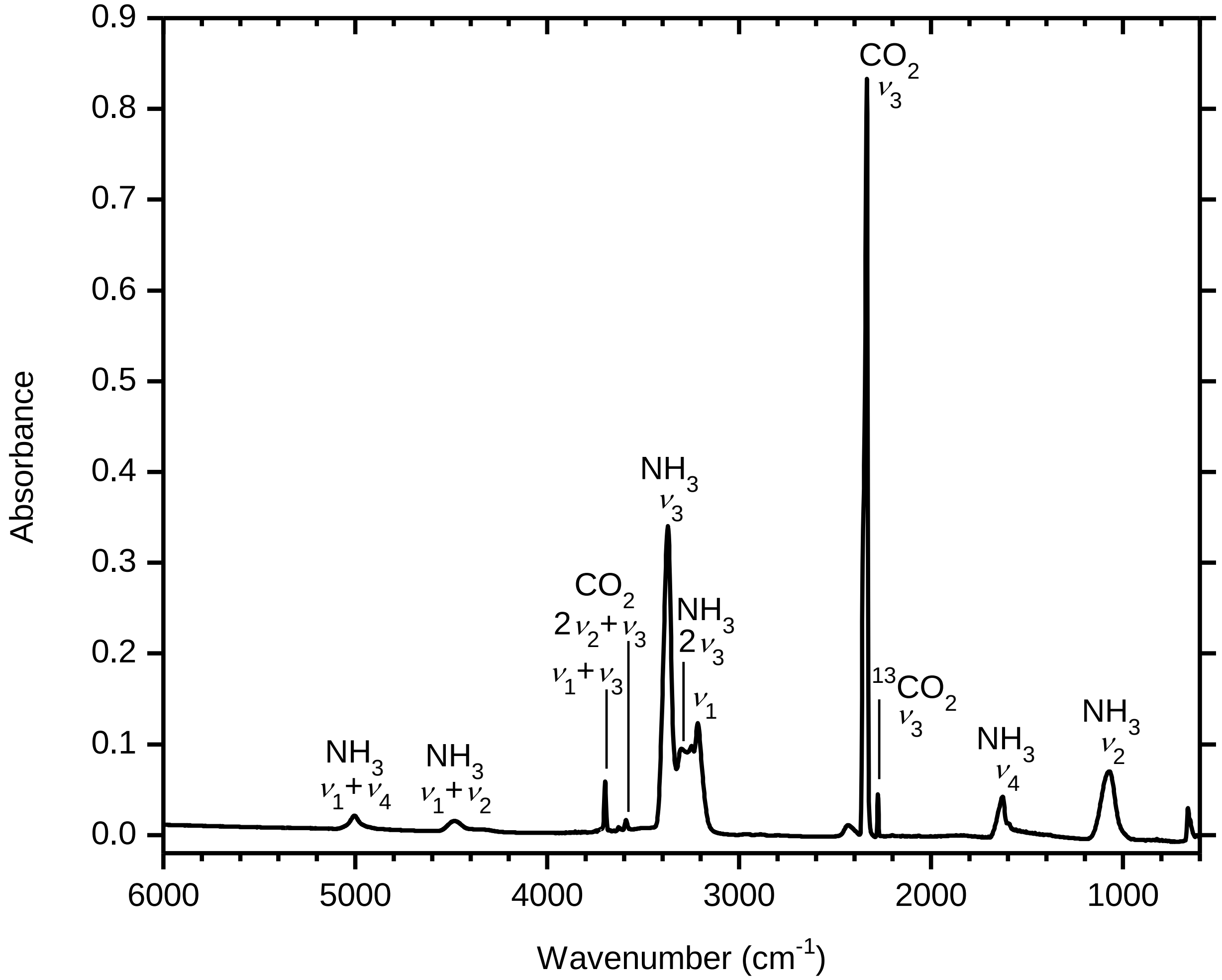

Figure S1. Infrared spectrum of freshly deposited ammonia – carbon dioxide ice.

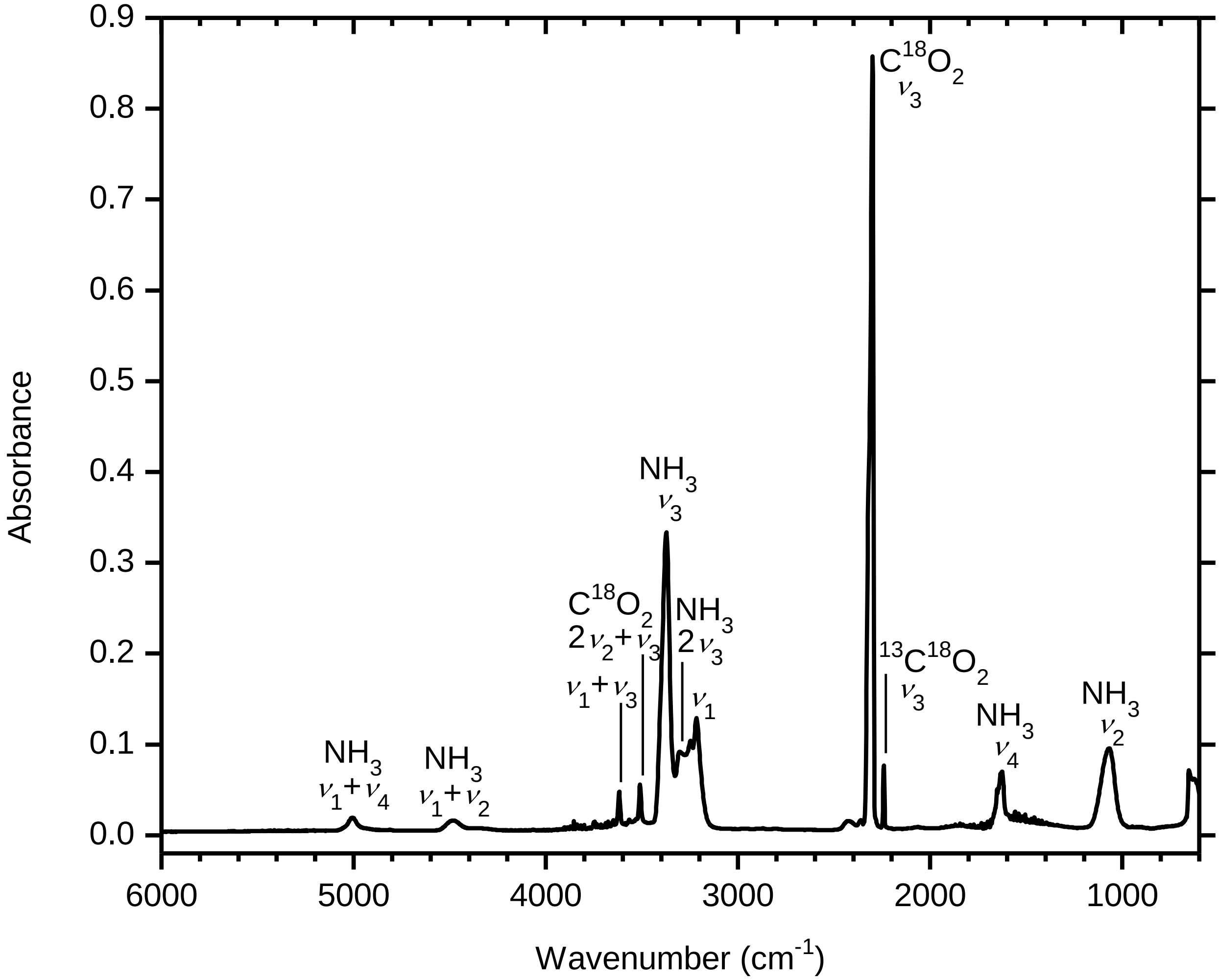

Figure S2. Infrared spectrum of freshly deposited ammonia – carbon dioxide-$C^{18}O_2$ ice.

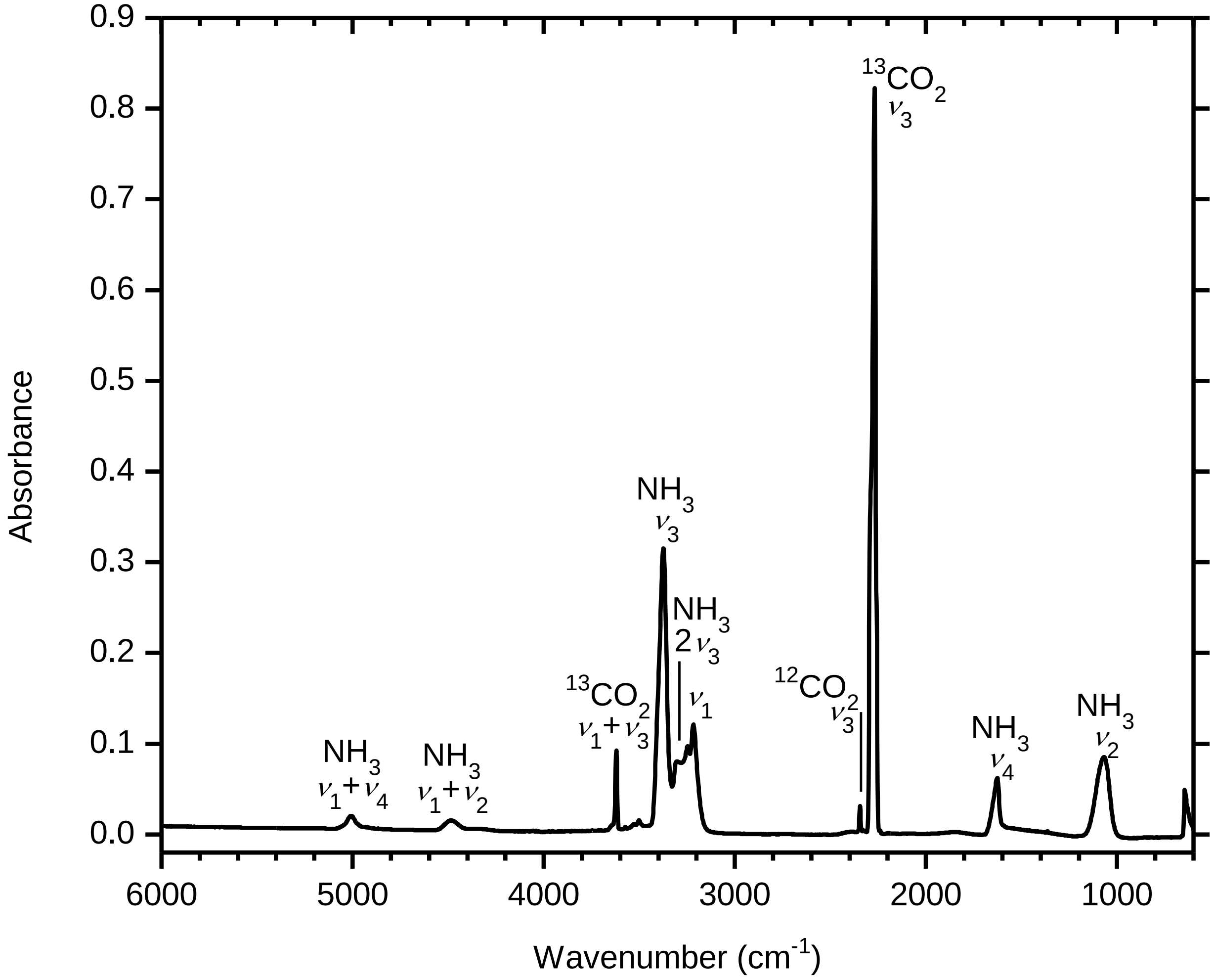

Figure S3. Infrared spectrum of freshly deposited ammonia – carbon dioxide-$^{13}CO_2$ ice.

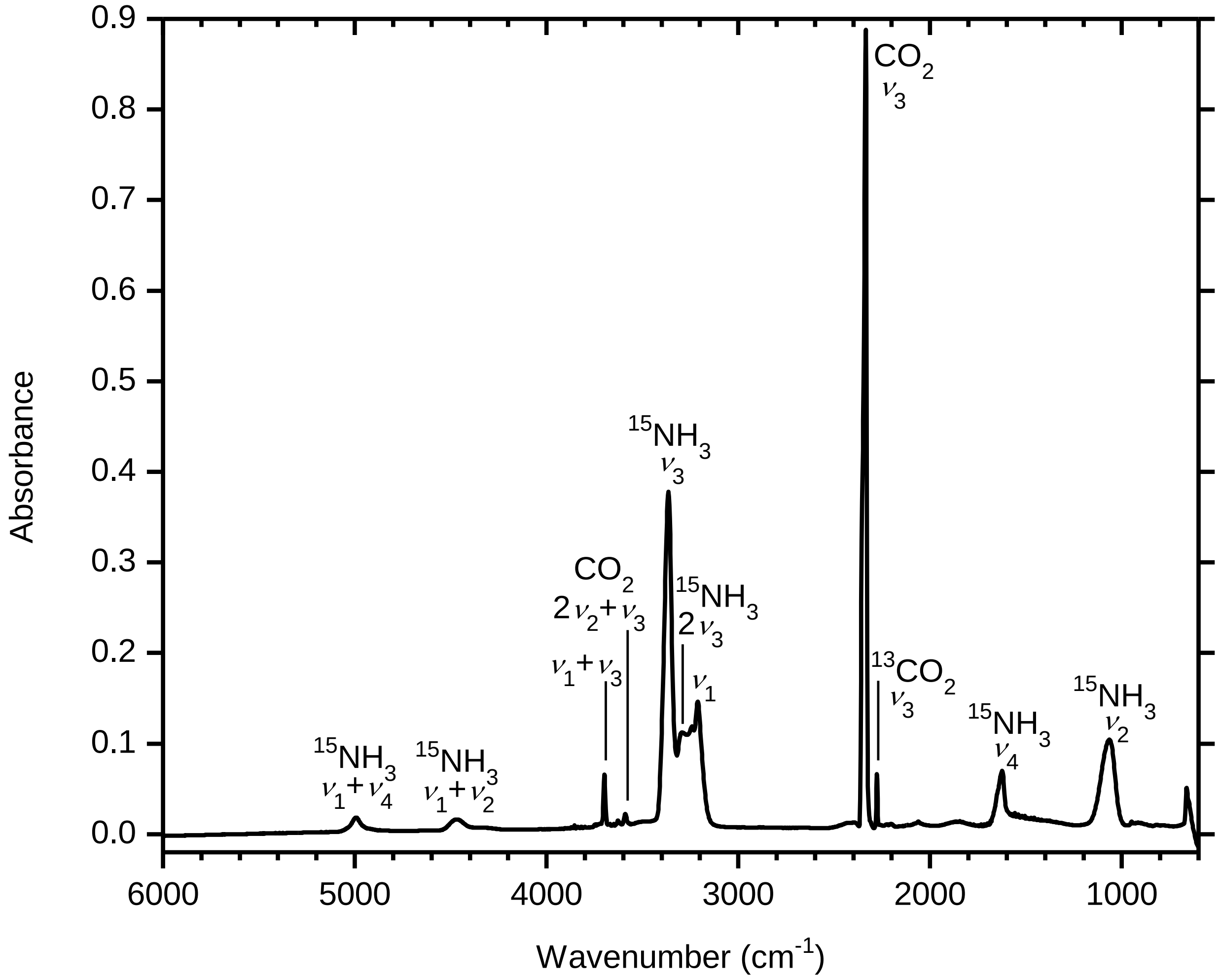

Figure S4. Infrared spectrum of freshly deposited ammonia-$^{15}NH_3$ – carbon dioxide ice.

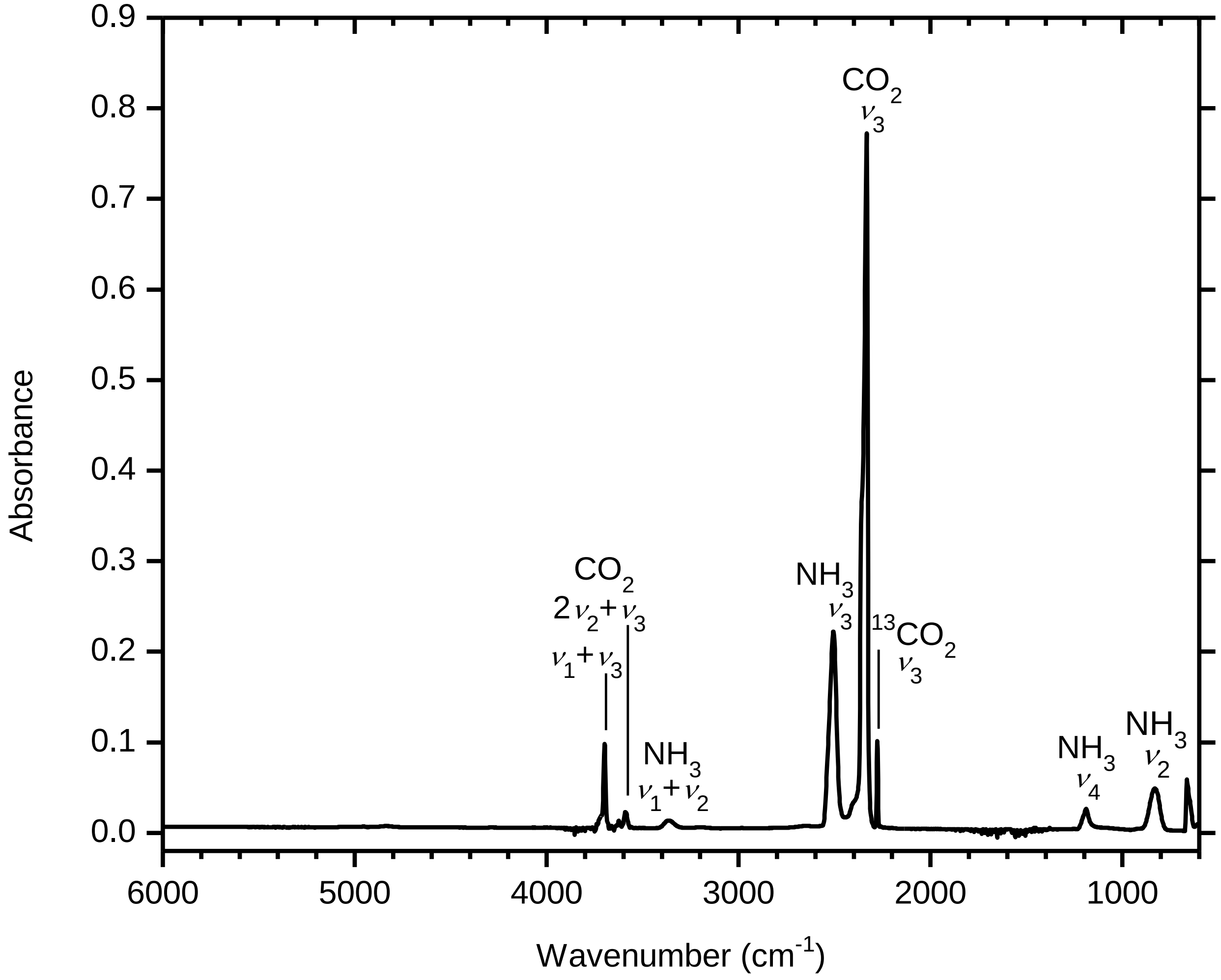

Figure S5. Infrared spectrum of freshly deposited ammonia-ND_3 – carbon dioxide ice.

Table S1. Ices studied in this experiment containing ammonia and carbon dioxide or their isotopomers. The ratio of the concentration of the components, ice thickness, and photon energy used are listed.

Experiment	Composition	Ratio	Thickness (nm)	Photon energy (eV)
1	CO_2:NH_3	1.2 ± 0.5 : 1	750 ± 50	11.10
2[1]	CO_2:NH_3	1.2 ± 0.5 : 1	750 ± 50	11.10
3	CO_2:NH_3	1.2 ± 0.5 : 1	750 ± 50	10.25
4[1]	CO_2:NH_3	1.2 ± 0.5 : 1	750 ± 50	10.25
5	CO_2:NH_3	1.5 ± 0.5 : 1	750 ± 50	8.45
6	$C^{18}O_2$:NH_3	1.0 ± 0.5 : 1	750 ± 50	10.25
7	CO_2:$^{15}NH_3$	1.2 ± 0.5 : 1	750 ± 50	10.25
8	$^{13}CO_2$:NH_3	1.1 ± 0.5 : 1	750 ± 50	10.25
9	CO_2:ND_3	1.4 ± 0.5 : 1	750 ± 50	10.25
10	$^{13}CO_2$:NH_3	1.2 ± 0.5 : 1	750 ± 50	11.10
11	$C^{18}O_2$:NH_3	1.1 ± 0.5 : 1	750 ± 50	11.10
12	CO_2:$^{15}NH_3$	1.3 ± 0.5 : 1	750 ± 50	11.10
13	CO_2:ND_3	1.2 ± 0.5 : 1	750 ± 50	11.10
14	CO_2:$^{15}NH_3$	1.2 ± 0.5 : 1	750 ± 50	–
15	CO_2:NH_3	1.7 ± 0.5 : 1	750 ± 50	–
16	$C^{18}O_2$:NH_3	1.4 ± 0.5 : 1	750 ± 50	–

[1]Irradiated for 30 minutes with 50 nA of 5 keV electrons

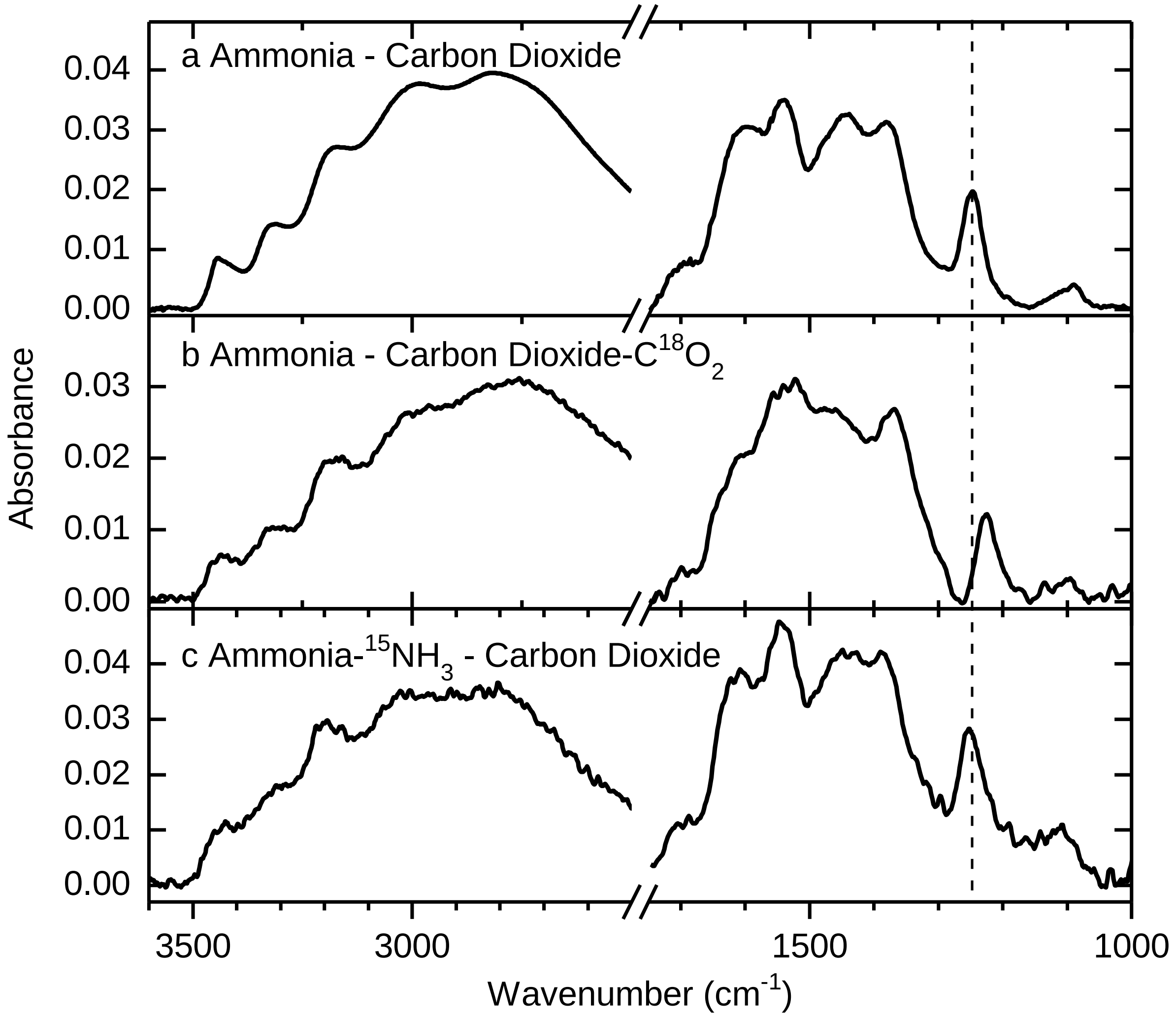

Figure S6. Infrared spectra at 280 K of (a) ammonia – carbon dioxide, (b) ammonia – carbon dioxide-$C^{18}O_2$, and (c) ammonia-$^{15}NH_3$ – carbon dioxide ices. The dashed vertical line indicates the position (1247 cm^{-1}) of the C–O stretch without isotopic labeling.

Table S2. Positions of peaks observed in ammonia – carbon dioxide, ammonia – carbon dioxide-$C^{18}O_2$, and ammonia-$^{15}NH_3$ – carbon dioxide ices, and likely assignments.[21]

Ammonia – carbon dioxide	ammonia – carbon dioxide-$C^{18}O_2$	ammonia-$^{15}NH_3$ – carbon dioxide	Assignment
3442	3430	3438	ν_{as} NH_2
3313	3310	3308	ν_{as} NH_2
3173	3174	3193	ν_s NH_2
2981	2988	2998	ν_s NH_2
2814	2759	2799	ν_s NH_2
1688	1699	1697	
1596	1606	1613	
1542	1533	1545	
1444	1465	1444	ν CO
1380	1371	1390	δ NH_2
1247	1228	1249	ν C–O
1091	1096	1112	ρ NH_2
820	815	818	τ NH_2

Table S3. Carbamic acid dimer zero-point corrected electronic energy (E at 0 K) and ionization energy (IE) at the CCSD(T)/CBS level with zero-point vibrational energy, Cartesian coordinates (Angstrom), vibrational frequencies, and vibrational intensities calculated at the B3LYP/cc-pVTZ level.

E[CCSD(T)/CBS] = -489.876639 Ha
IE[CCSD(T)/CBS] = 9.95 eV
E[B3LYP/cc-pVTZ] = -490.416153 Ha
ZPVE[B3LYP/cc-pVTZ] = 0.103725 Ha

Atom	x	y	z
H	1.263849	3.61486	0.047066
H	2.735638	2.687153	0.050628
H	1.146184	-0.310904	-0.000233
H	-1.263849	-3.61486	0.047066
H	-2.735638	-2.687153	0.050628
H	-1.146184	0.310904	-0.000233
C	0.997909	1.600105	-0.000932
C	-0.997909	-1.600105	-0.000932
N	1.736091	2.733164	-0.013764
N	-1.736091	-2.733164	-0.013764
O	-0.235222	1.625514	0.000208
O	1.736091	0.504919	0.000353
O	0.235222	-1.625514	0.000208
O	-1.736091	-0.504919	0.000353

Frequency (cm^{-1})	Intensity (km mol^{-1})	Frequency (cm^{-1})	Intensity (km mol^{-1})
50.8857	1.4487	1013.5385	22.0766
67.7513	1.137	1023.2393	0.0732
97.1206	17.0352	1100.6955	0.0147
113.6713	413.6448	1105.1725	15.2638
114.2483	2.7397	1380.231	0.0012
172.055	0.3877	1388.4665	608.7929
198.3018	0.3608	1506.7698	125.1948
211.6932	48.4244	1527.8616	0.0367
507.6482	0.0008	1600.0454	0.5622
545.5912	44.2089	1623.7866	382.562
557.9816	0.3044	1727.0474	0.0977
560.7513	29.0695	1758.8305	1246.383
636.5226	0.0161	2934.2387	0.0001
655.634	22.563	3062.4273	3909.3742
791.7295	0.0073	3621.2851	96.0524
793.4902	15.9592	3621.6559	0.5494
952.9947	0.0093	3755.803	0.0008
994.9402	178.0167	3755.8575	124.7305

Table S4. Carbamic acid dimer cation zero-point corrected electronic energy (E at 0 K) at the CCSD(T)/CBS level with electronic energy, zero-point vibrational energy, Cartesian coordinates (Angstrom), vibrational frequencies, and vibrational intensities calculated at the B3LYP/cc-pVTZ level.

E[CCSD(T)/CBS] = -489.511458 Ha
E[B3LYP/cc-pVTZ] = -490.071349 Ha
ZPVE[B3LYP/cc-pVTZ] = 0.104119 Ha

Atom	x	y	z
H	-1.78149	3.048982	-0.016486
H	-0.251436	3.88136	0.004823
H	1.540431	1.027581	0.015268
H	1.78149	-3.048982	-0.016486
H	0.251436	-3.88136	0.004823
H	-1.540431	-1.027581	0.015268
C	-0.136864	1.86489	-0.000442
C	0.136864	-1.86489	-0.000442
N	-0.775633	3.02012	-0.004497
N	0.775633	-3.02012	-0.004497
O	-0.775633	0.767204	-0.010206
O	1.169849	1.925768	0.014021
O	0.775633	-0.767204	-0.010206
O	-1.169849	-1.925768	0.014021

Frequency (cm^{-1})	Intensity (km mol^{-1})	Frequency (cm^{-1})	Intensity (km mol^{-1})
4.2978	0.5778	1037.5168	16.034
41.4509	6.4057	1042.18	0.003
56.772	0.0022	1086.4906	0.0015
104.0416	2.2176	1087.5057	33.745
181.641	0.0004	1241.2907	0.033
230.221	0.0001	1242.1235	334.6915
435.8541	386.2823	1493.0037	1349.3464
437.3397	0.072	1523.5077	0.0412
481.8176	14.6827	1571.6869	599.3849
492.7309	0.2245	1609.939	0.0282
496.3874	28.8834	1672.7205	1332.88
505.8405	0.0177	1706.2203	0.001
579.0743	73.9273	3570.4787	562.3039
629.0433	0.0278	3573.7887	0.0009
633.628	0.2329	3687.8425	84.2785
638.0424	249.64	3691.56	0.0102
759.4192	0.0064	3698.3781	0.0383
760.865	45.3826	3698.3927	273.4669

Table S5. Carbamic acid-^{18}O dimer electronic energy (E at 0 K), zero-point vibrational energy, Cartesian coordinates (Angstrom), vibrational frequencies, and vibrational intensities calculated at the B3LYP/cc-pVTZ level.

E[B3LYP/cc-pVTZ] = -490.416891 Ha
ZPVE[B3LYP/cc-pVTZ] = 0.102987 Ha

Atom	x	y	z
H	1.263849	3.61486	0.047066
H	2.735638	2.687153	0.050628
H	1.146184	-0.310904	-0.000233
H	-1.263849	-3.61486	0.047066
H	-2.735638	-2.687153	0.050628
H	-1.146184	0.310904	-0.000233
C	0.997909	1.600105	-0.000932
C	-0.997909	-1.600105	-0.000932
N	1.736091	2.733164	-0.013764
N	-1.736091	-2.733164	-0.013764
O	-0.235222	1.625514	0.000208
O	1.736091	0.504919	0.000353
O	0.235222	-1.625514	0.000208
O	-1.736091	-0.504919	0.000353

Frequency (cm^{-1})	Intensity (km mol^{-1})	Frequency (cm^{-1})	Intensity (km mol^{-1})
48.8814	1.2481	990.8892	14.6074
65.4794	1.2425	992.2254	159.2529
96.4622	15.5979	1088.4114	0.0209
110.722	4.2334	1093.1184	19.2625
113.6577	413.4247	1367.7198	0.001
163.978	0.4514	1377.0277	595.7977
192.6589	0.4094	1501.4902	132.4797
205.8197	46.2489	1514.5376	0.0189
495.164	0.002	1594.6686	0.557
532.553	57.933	1620.7178	346.9901
555.7353	7.2143	1716.0016	0.1239
556.8398	0.3287	1735.4544	1232.4391
611.3526	0.0214	2922.7937	0.0001
630.9656	18.7882	3051.8419	3883.2332
785.1798	0.0047	3621.2727	96.9618
787.5808	16.9633	3621.6514	0.5494
951.4123	0.0075	3755.802	0.0008
980.1009	22.8199	3755.8562	124.8583

Table S6. Carbamic acid-^{18}O dimer cation electronic energy (E at 0 K), zero-point vibrational energy, Cartesian coordinates (Angstrom), vibrational frequencies, and vibrational intensities calculated at the B3LYP/cc-pVTZ level.

E[B3LYP/cc-pVTZ] = -490.072049 Ha
ZPVE[B3LYP/cc-pVTZ] = 0.103419 Ha

Atom	x	y	z
H	1.263849	3.61486	0.047066
H	2.735638	2.687153	0.050628
H	1.146184	-0.310904	-0.000233
H	-1.263849	-3.61486	0.047066
H	-2.735638	-2.687153	0.050628
H	-1.146184	0.310904	-0.000233
C	0.997909	1.600105	-0.000932
C	-0.997909	-1.600105	-0.000932
N	1.736091	2.733164	-0.013764
N	-1.736091	-2.733164	-0.013764
O	-0.235222	1.625514	0.000208
O	1.736091	0.504919	0.000353
O	0.235222	-1.625514	0.000208
O	-1.736091	-0.504919	0.000353

Frequency (cm^{-1})	Intensity (km mol^{-1})	Frequency (cm^{-1})	Intensity (km mol^{-1})
4.085	0.4746	999.6222	26.9413
40.5708	6.656	1004.0018	0.0042
54.3315	0.0023	1075.0691	0.0019
100.456	2.3048	1076.6834	42.1679
176.1115	0.0004	1229.5974	0.0322
220.9762	0	1229.7495	334.6912
435.7497	385.5529	1474.2914	1383.8445
437.3374	0.0735	1511.5016	0.0443
479.7903	15.4488	1566.6849	547.1885
484.7111	26.7025	1602.9093	0.0235
490.8876	0.024	1670.8194	1275.3109
493.5723	0.0298	1700.7654	0.0004
558.4246	68.6366	3570.4671	563.1247
606.5276	0.0036	3573.781	0.0009
628.448	0.0301	3676.0186	79.537
636.8712	253.5439	3679.9007	0.0059
753.5556	0.0058	3698.2351	0.0425
755.0077	40.2804	3698.3854	274.7965

Table S7. Four-wave mixing schemes were employed to generate vacuum ultraviolet (VUV) photons for photoionization in experiments 1–13 (Table S1). All experiments use at least one dye laser (Sirah Lasertechnik, Cobra-Stretch) pumped by a neodymium yttrium-aluminum garnet (Nd:YAG, Spectra-Physics, Quanta Ray PRO 270-30 or PRO 250-30) laser harmonic (355 or 532 nm) appropriate for the dye in use. Because of the orientation of the FTIR and mass spectrometric probes in the experimental chamber, only one may be measured during TPD, these experiments are those for which the PI-ReToF-MS technique was used.

Experiments	Medium	ω_{VUV} =	ω_1 (nm)	ω_1 Dye	ω_2 (nm)	ω_2 Dye	Energy (eV)
1, 8–13	Xenon	$2\omega_1 + \omega_2$	249.628	Coumarin 503	1064[a]	-	11.10
2, 4–7	Krypton	$2\omega_1 - \omega_2$	202.316	Rhodamine 610/640	617.9	DCM	10.25
3	Xenon	$2\omega_1 - \omega_2$	222.566	Coumarin 450	460.7	Coumarin 460	8.45

[a] Nd:YAG harmonic

Table S8. Parameters used in irradiation dose calculation and resulting doses.

Irradiated area	1.6 ± 0.1 cm^2
Initial kinetic energy of e^-	5.000 keV
Irradiation current	50 nA
Total number of e^-	(5.6 ± 0.06) × 10^{14}
Total molecules irradiated	9.58 × 10^{17}
Approximate density of mixed ice[8]	0.92_5 g cm^{-1}
Average Penetration depth	325 ± 10 nm
Fraction of backscattered energy	0.241
Fraction of Transmitted e^-	0.000
Dose per molecule of ammonia	1.24 ± 0.03
Dose per molecule of carbon dioxide	3.2 ± 0.1

References

(1) Abplanalp, M. J.; Förstel, M.; Kaiser, R. I. Exploiting Single Photon Vacuum Ultraviolet Photoionization to Unravel the Synthesis of Complex Organic Molecules in Interstellar Ices. *Chem. Phys. Lett.* **2016**, *644*, 79-98.

(2) Abplanalp, M. J.; Gozem, S.; Krylov, A. I.; Shingledecker, C. N.; Herbst, E.; Kaiser, R. I. A Study of Interstellar Aldehydes and Enols as Tracers of a Cosmic Ray-Driven Nonequilibrium Synthesis of Complex Organic Molecules. *Proc. Natl. Acad. Sci. U.S.A.* **2016**, *113*, 7727-7732.

(3) Jones, B. M.; Kaiser, R. I. Application of Reflectron Time-of-Flight Mass Spectroscopy in the Analysis of Astrophysically Relevant Ices Exposed to Ionization Radiation: Methane (CH_4) and D_4-Methane (CD_4) as a Case Study. *J. Phys. Chem. Lett.* **2013**, *4*, 1965-1971.

(4) Maity, S.; Kaiser, R. I.; Jones, B. M. Infrared and Reflectron Time-of-Flight Mass Spectroscopic Study on the Synthesis of Glycolaldehyde in Methanol (CH_3OH) and Methanol–Carbon Monoxide (CH_3OH–CO) Ices Exposed to Ionization Radiation. *Faraday Discuss.* **2014**, *168*, 485-516.

(5) Turner, A. M.; Abplanalp, M. J.; Chen, S. Y.; Chen, Y. T.; Chang, A. H. H.; Kaiser, R. I. A Photoionization Mass Spectroscopic Study on the Formation of Phosphanes in Low Temperature Phosphine Ices. *Phys. Chem. Chem. Phys.* **2015**, *17*, 27281-27291.

(6) Hudson, R. L.; Yarnall, Y. Y.; Gerakines, P. A. Infrared Spectral Intensities of Amine Ices, Precursors to Amino Acids. *Astrobiol.* **2022**, *22*, 452-461.

(7) Hudson, R. L.; Coleman, F. M. Infrared Intensities and Molar Refraction of Amorphous Dimethyl Carbonate – Comparisons to Four Interstellar Molecules. *Phys. Chem. Chem. Phys.* **2019**, *21*, 11284-11289.

(8) Bouilloud, M.; Fray, N.; Bénilan, Y.; Cottin, H.; Gazeau, M. C.; Jolly, A. Bibliographic Review and New Measurements of the Infrared Band Strengths of Pure Molecules at 25 K: H_2O, CO_2, CO, CH_4, NH_3, CH_3OH, HCOOH and H_2CO. *Mon. Not. R. Astron. Soc.* **2015**, *451*, 2145-2160.

(9) Becke, A. D. Density-Functional Thermochemistry. I. The Effect of the Exchange-Only Gradient Correction. *J. Chem. Phys.* **1992**, *96*, 2155-2160.

(10) Becke, A. D. Density-Functional Thermochemistry. II. The Effect of The Perdew–Wang Generalized-Gradient Correlation Correction. *J. Chem. Phys.* **1992**, *97*, 9173-9177.

(11) Becke, A. D. Density Functional Thermochemistry. III. The Role of Exact Exchange. *J. Chem. Phys.* **1993**, *98*, 5648-5652.

(12) Lee, C.; Yang, W.; Parr, R. G. Development of the Colle-Salvetti Correlation-Energy Formula into a Functional of the Electron Density. *Phys. Rev. B* **1988**, *37*, 785-789.

(13) Deegan, M. J. O.; Knowles, P. J. Perturbative Corrections to Account for Triple Excitations in Closed and Open Shell Coupled Cluster Theories. *Chem. Phys. Lett.* **1994**, *227*, 321-326.

(14) Hampel, C.; Peterson, K. A.; Werner, H.-J. A Comparison of the Efficiency and Accuracy of the Quadratic Configuration Interaction (QCISD), Coupled Cluster (CCSD), and Brueckner Coupled Cluster (BCCD) Methods. *Chem. Phys. Lett.* **1992**, *190*, 1-12.

(15) Knowles, P. J.; Hampel, C.; Werner, H.-J. Coupled Cluster Theory for High Spin, Open Shell Reference Wave Functions. *J. Chem. Phys.* **1993**, *99*, 5219-5227.

(16) Purvis, G. D.; Bartlett, R. J. A Full Coupled-Cluster Singles and Doubles Model: The Inclusion of Disconnected Triples. *J. Chem. Phys.* **1982**, *76*, 1910-1918.

(17) Peterson, K. A.; Woon, D. E.; Dunning, T. H. Benchmark Calculations with Correlated Molecular Wave Functions. IV. The Classical Barrier Height of the $H+H_2 \rightarrow H_2+H$ Reaction. *J. Chem. Phys.* **1994**, *100*, 7410-7415.

(18) Zhu, C.; Frigge, R.; Turner, A. M.; Kaiser, R. I.; Sun, B. J.; Chen, S. Y.; Chang, A. H. H. First Identification of Unstable Phosphino Formic Acid (H_2PCOOH). *Chem. Commun.* **2018**, *54*, 5716-5719.

(19) Frigge, R.; Zhu, C.; Turner, A. M.; Abplanalp, M. J.; Sun, B. J.; Huang, Y. S.; Chang, A. H. H.; Kaiser, R. I. Synthesis of the Hitherto Elusive Formylphosphine ($HCOPH_2$) in the Interstellar Medium - A Molecule with an Exotic Phosphorus Peptide Bond. *Chem. Commun.* **2018**, *54*, 10152-10155.

(20) Frisch, M. J.; Trucks, G. W.; Schlegel, H. B.; Scuseria, G. E.; Robb, M. A.; Cheeseman, J. R.; Scalmani, G.; Barone, V; Petersson, G. A.; Nakatsuji, H.; et al. *Gaussian 16*, Revision C,01; Gaussian, Inc.: Wallingford, CT, 2016.

(21) Rodriguez-Lazcano, Y.; Mate, B.; Herrero, V. J.; Escribano, R.; Galvez, O. The Formation of Carbamate Ions in Interstellar Ice Analogues. *Phys. Chem. Chem. Phys.* **2014**, *16*, 3371-3380.
