# Supplementary material for: Thermal Synthesis of Carbamic Acid and Its Dimer in Interstellar Ices: A Reservoir of Interstellar Amino Acids
Source: ACS Cent Sci. 2023 Nov 29;9(12):2241–50. doi: 10.1021/acscentsci.3c01108 (PMC10755733; doi:10.1021/acscentsci.3c01108)
Supplement: Supplementary file 2 — oc3c01108_si_002.pdf [file oc3c01108_si_002.pdf]

oc-2023-01108t.R1

Name: Peer Review Information for "Thermal Synthesis of Carbamic Acid and its Dimer in Interstellar Ices — A Reservoir of Interstellar Amino Acids"

First Round of Reviewer Comments

Reviewer: 1

Comments to the Author

REVIEW ON THE MANUSCRIPT oc-2023-01108t

Thermal Synthesis of Carbamic Acid and its Dimer in Interstellar Ices - A Reservoir of Interstellar Amino Acids

Joshua H. Marks, Jia Wang, Bing-Jian Sun, Mason McAnally, Andrew M. Turner, Agnes H.-H. Chang, Ralf I. Kaiser

General Comments

The submitted manuscript presents an experimental study of the thermal synthesis of carbamic acid and its dimer and ammonium carbamate in NH_3-CO_2 ices (T=10-320K). The detection of these species was carried out by combining two complementary detection techniques: FTIR (for the condensed-phase) and photoionization reflectron time-of-flight mass spectrometry (for the gas-phase), which provide valuable information that will certainly improve the knowledge of the synthesis of precursor of prebiotic molecules in the ISM and its mechanisms.

The paper is well written, structured, and very concise. However, some parts can be improved to aid the reader to fully understand what it was done. The discussion and the conclusions arose from this study are plausible. Therefore, I strongly recommend the publication of the present paper in *ACS Central Science,* once the specific comments are addressed by the authors.

Specific comments

Title: Why ammonium carbamate is not included in the title?

Graphical abstract: I suggest including the molecular formula and name below the chemical structure.

Abstract: Carbamic acid could be seen as both an amine and carboxylic acid, and therefore an amino acid; however, the attachment of the carboxyl group –COOH directly to the nitrogen atom (without any intermediate carbon chain) makes it behave very differently from the amino acids with intermediate carbon chain. So, I do not call carbamic acid the simplest amino acid. Glycine is considered the simplest amino acid.

Introduction

On Page 5, it is stated that chemistry on "*ices can result in substituted carbamates which are thermally stable and serve as key **biochemical** molecules*.[10]" Please, check if reference 10 is a good reference for that statement. It may refer to the thermal stability of (alkyl)substituted carbamates (in solution? Solid?), but nothing is said about serving as key biochemical molecules. Do the authors mean "as key precursor of biochemical molecules". In biochemistry, carbamates are observed as protein or amino acid residues. In the interstellar medium carbamates, like methyl carbamate, are candidate precursors of biological molecules.

Calculations of the IR absorption spectrum of carbamic acid and ammonium carbamate

The authors state on Page 6 that although the calculations of the integrated intensities are for the gas phase, they are in "near agreement" with those for the condensed phase. How "near" is the agreement?

What temperature was used in the calculations shown in Tables S2S5? This needs some clarification to avoid any confusion. The experiments were performed between 10 K and 320 K.

Temperature-resolved IR absorption of carbamic acid and ammonium carbamate

I was initially a bit confused with Figure 4. Data shown in this figure are experimental, but as these results are just after the calculations, it can be confusing for the reader. In fact, the explanation of what is plotted in Figure 4 is not given until Page 9." *Changes in the **relative abundance** of these two species within the ice are measured by the changes in **intensities of the molecule-specific vibrational absorptions**".* That answered one of my questions, when reading: Does the "normalized intensity" refer to the "absorbance"? As you know, the "integrated intensities" are independent of concentration of the absorbing species, while the "absorbances" are dependent on concentration. This figure agrees with the experimental results presented in Figure 3, if intensity refers to absorbance. However, (Page 7, Line 4) it is stated "*The temperature-resolved **integrated intensity** of infrared absorptions*…". In addition, the discussion on Page 7 is centered in *abundances and depletion/formation processes*, which may refer to concentrations (i.e., absorbances). Further details are needed to better understand what is presented.

Please, clarify this aspect.

Figures

Figure 3 caption. The colors of the spectra in panels b and c do not match with the text in the caption.

"*At (b) 200 K peaks assigned to ammonium carbamate (2, dark red) and carbamic acid (1, green) are observed"* There is no spectrum in green. In the legend the dark red spectra are for both the ammonium carbamate and carbamic acid. However, there are two dark red spectra. Please, correct.

"At (c) 280 K the peaks assigned to carbamic acid are more pronounced and a peak assigned here to its dimer (magenta) is observed in the C-O stretching region." In the figure, the IR spectrum in green, not magenta, is assigned to the dimer. Please, correct.

Figure 5 caption. X-axis in panels c and d should be "temperature", right?

Supporting information

Figure S2 caption: "ammonia–carbon dioxide-$^{18}O_2$ ice" can be interpreted that the ice also contains isotopic molecular oxygen. Please replace by "$C^{18}O_2$" or "NH_3-$C^{18}O_2$". The same for Figures S3-S6. As a reader, I prefer reading the ices as presented in Table S1.

Figures S1-S5 are the IR spectrum for freshly prepared ices T=5–10 K. Were these IR spectra recorded as a function of temperature as done for NH_3-CO_2 ices? What is the purpose of studying the IR spectrum of the isotopically labelled ices? Just to check the red-shift of the absorption bands?

Table S6: Which are experiments 1 – 11?

Minor changes

Throughout the manuscript: Starting a sentence with the number assigned to the carbamic acid (**1**) or ammonium carbamate (**2**) is odd. In those cases, I suggest using the molecular formula. Perhaps the use of the numbers in the text can be reduced.

Page 5, Lines 5 and 34*:* Ultraviolet (UV) is defined on page 3. It can be written as "…by the intense UV and vacuum UV…" since the acronym VUV does not longer appear in the text. The same for the acronym "UHV" (although is repeated in the SI).

Page 6, Line 35: "carbon dioxide-ammonia-^{15}N and carbon dioxide-$^{18}O_2$ammonia" can be written as "CO_2-$^{15}NH_3$ and $C^{18}O_2$– NH_3. The red-shift of 19 cm^{-1} in spectrum B of Figure S6 is not clearly seen. Maybe if spectrum B is overlapped with A the shift can be perceptible (or not since it is a small shift). Another way of presenting Figure S6 is desired.

Page 9, Lines 11 and 24: Parenthesis in NH_3 has to be removed: 2 NH_3.

Page 9, Lines 24 and 25: Reactions [5] and [6] can be numbered as [-3] and [-1] since it is the reverse reaction of [3] and [1], respectively.

Reviewer: 2

Comments to the Author

This is a well written and clearly presented paper with an excellent review of previous work. The authors analysis and conclusions are sound. I see no obvious additions required.

Reviewer: 3

Comments to the Author

This manuscript reports on the experimental investigation of the formation and interconversion pathways of carbamic acid and ammonium carbamate along with the dimerization of the former in binary carbon dioxide-ammonia interstellar analog ices. Temperature-resolved condensed-phase FTIR spectroscopy was combined with gas-phase detection via isomer-selective single-photon photoionization time-of-flight mass spectrometry. This study is carried out in experimental conditions that mimic those of the interstellar medium (both in terms of temperature and density) and thus provides an important evidence that these prebiotic molecules can be formed in interstellar ices without energetic radiation. The manuscript is well and clearly written. The conclusions are well sounded and the discussion is well argued. Therefore, in my opinion is deserved to be published.

This study provides an important piece of the origin-of-life puzzle by demonstrating that carbamic acid and ammonium carbamate can be produced in the interstellar medium. I have however a concern about the ice composition. In the interstellar medium, this is expected to be mainly formed by water with other molecules in less abundance. How much does the reaction mechanism rely on the only presence of the two reactants? Would the vibrational features be affected by a water-ice environment and how much?

Author's Response to Peer Review Comments:

Editorial Comments:

AU EMAIL: Please label as "email"

This has been corrected.

SYNOPSIS MISSING: The synopsis should be no more than 200 characters (including spaces) and should reasonably correlate with the TOC graphic. The synopsis is intended to explain the importance of the article to a broader readership across the sciences. Please place your synopsis in the manuscript file after the TOC graphic, and label it as "Synopsis." The following synopsis has been added:
"The carbamic acid dimer forms and remains in model interstellar ice at temperatures up to 290 K. Near young stellar objects it would provide a feedstock for amino acids needed for the origins of life."

SI PG#S: The supporting information pages must be numbered consecutively, starting with page S1.
Page numbers have been added to the supporting information.

Reviewer: 1

Recommendation: Publish in ACS Central Science after minor revisions noted.

Comments: See attached file.

General Comments
The submitted manuscript presents an experimental study of the thermal synthesis of carbamic acid and its dimer and ammonium carbamate in NH3-CO2 ices (T=10-320K). The detection of these species was carried out by combining two complementary detection techniques: FTIR (for the condensed-phase) and photoionization reflectron time-of-flight mass spectrometry (for the gasphase), which provide valuable information that will certainly improve the knowledge of the synthesis of precursor of prebiotic molecules in the ISM and its mechanisms.

The paper is well written, structured, and very concise. However, some parts can be improved to aid the reader to fully understand what it was done. The discussion and the conclusions arose from this study are plausible. Therefore, I strongly recommend the publication of the present paper in *ACS Central Science,* once the specific comments are addressed by the authors. We thank the reviewer for their thorough analysis of this manuscript.

Specific comments
Title: Why ammonium carbamate is not included in the title?
The dimerization of carbamic acid and the resulting thermal stability are the major findings of this research. The presence of ammonium carbamate is relevant insofar as it decomposes to yield carbamic acid, but its presence and decomposition are not new findings, and we would prefer to emphasize the role of carbamic acid.

Graphical abstract: I suggest including the molecular formula and name below the chemical structure.

Molecular formulas and names have been added to the Graphical Abstract.

Abstract: Carbamic acid could be seen as both an amine and carboxylic acid, and therefore an amino acid; however, the attachment of the carboxyl group –COOH directly to the nitrogen atom (without any intermediate carbon chain) makes it behave very differently from the amino acids with intermediate carbon chain. So, I do not call carbamic acid the simplest amino acid. Glycine is considered the simplest amino acid.

The section of the abstract which read:

"Carbamic acid (H_2NCOOH) contains amino (–NH_2) and carboxylic acid (–COOH) groups and hence is the simplest amino acid. When present in interstellar ices, it serves as a condensed-phase

source of the molecular building blocks for more complex proteinogenic amino acids." Has been modified to read:
"When present in interstellar ices, carbamic acid (H_2NCOOH) can serve as a condensed-phase source of the molecular building blocks for more complex proteinogenic amino acids. When present in interstellar ices, carbamic acid (H_2NCOOH) can serve as a condensed-phase source of

the molecular building blocks for more complex proteinogenic amino acids." And in the introduction where this was restated we have changed the text: "Carbamic acid (H_2NCOOH, **1**) represents the simplest amino acid"
Which now reads:

"Carbamic acid (H_2NCOOH, **1**) might be considered the simplest amino acid, though the lack of an additional carbon between the carboxyl and amino groups gives it a different chemistry than proteinogenic amino acids ($H_2NCH(R)COOH$)."

Introduction

On Page 5, it is stated that chemistry on "*ices can result in substituted carbamates which are thermally stable and serve as key* ***biochemical*** *molecules*.10" Please, check if reference 10 is a good reference for that statement. It may refer to the thermal stability of (alkyl)substituted carbamates (in solution? Solid?), but nothing is said about serving as key biochemical molecules. Do the authors mean "as key precursor of biochemical molecules". In biochemistry, carbamates are observed as protein or amino acid residues. In the interstellar medium carbamates, like methyl

carbamate, are candidate precursors of biological molecules.
We have replaced this use of reference 10 with Ghosh & Brindisi (2015) which has a more specific discussion of the stability of carbamates in a biochemical environment. We have also changed the text to "…serve as precursors to key biochemical molecules".

Calculations of the IR absorption spectrum of carbamic acid and ammonium carbamate

The authors state on Page 6 that although the calculations of the integrated intensities are for the gas phase, they are in "near agreement" with those for the condensed phase. How "near" is the agreement?
The language used initially (below) was not sufficiently clear.

“Though these calculations make use of gas-phase B3LYP/cc-pVTZ methodology, they are in near agreement with conventional condensed-phase infrared analysis which predicts a C–O stretch for carboxylic acid dimers.[59]”

Our goal is to explain that the difference between the computed and observed band positions are very likely due to the comparison between gas-phase theory and condensed phase measurements. We have edited the text to reflect this:

“These calculations make use of gas-phase B3LYP/cc-pVTZ methodology, and the predicted wavelength without isotopic labeling is 95 cm^{-1} higher than the observed position. This is likely the result of a combination of anharmonicity and comparison between gas-phase calculations and condensed phase measurements, and the position of this vibrational mode is within the range in which other carboxylic acids have been found to vibrate.[59]”

What temperature was used in the calculations shown in Tables S2-S5? This needs some clarification to avoid any confusion. The experiments were performed between 10 K and 320 K. These calculations were all at 0 K, the text “(E at 0 K)” has been added to make this clear.

Temperature-resolved IR absorption of carbamic acid and ammonium carbamate

I was initially a bit confused with Figure 4. Data shown in this figure are experimental, but as these results are just after the calculations, it can be confusing for the reader. In fact, the explanation of what is plotted in Figure 4 is not given until Page 9.” *Changes in the **relative abundance** of these two species within the ice are measured by the changes in **intensities of the molecule-specific vibrational absorptions**”.* That answered one of my questions, when reading: Does the “normalized intensity” refer to the “absorbance”? As you know, the “integrated intensities” are independent of concentration of the absorbing species, while the “absorbances” are dependent on concentration. This figure agrees with the experimental results presented in Figure 3, if intensity refers to absorbance. However, (Page 7, Line 4) it is stated “*The temperature-resolved **integrated intensity** of infrared absorptions…*”. In addition, the discussion on Page 7 is centered in *abundances and depletion/formation processes*, which may refer to concentrations (i.e., absorbances). Further details are needed to better understand what is presented.

Please, Clarify this aspect.

The reviewer makes an excellent point, and the description of how this data was handled is lacking. To better clarify how we go from infrared spectra to the traces presented in Figure 4, we have included text with the introduction of Figure 4:

“The data presented in Figure 4 are temperature-dependent absorbance measurements of bands uniquely assigned to molecules within the ice. The resulting FTIR-TPD profiles are normalized to the same arbitrary maximum absorbance such that the relative rates of formation and the temperature-dependence thereof are apparent. The experimentally-observed temperature-resolved infrared absorbance of carbon dioxide…”

We also relabeled the vertical axes of Figure 4 to read more correctly “Absorbance (normalized)” rather than “Intensity (normalized)”.

Figures

Figure 3 caption. The colors of the spectra in panels b and c do not match with the text in the caption.

"*At (b) 200 K peaks assigned to ammonium carbamate (2, dark red) and carbamic acid (1, green) are observed*" There is no spectrum in green. In the legend the dark red spectra are for both the ammonium carbamate and carbamic acid. However, there are two dark red spectra. Please, correct. *"At (c) 280 K the peaks assigned to carbamic acid are more pronounced and a peak assigned here to its dimer (magenta) is observed in the C-O stretching region."* In the figure, the IR spectrum in green, not magenta, is assigned to the dimer. Please, correct.

Figure 5 caption. X-axis in panels c and d should be "temperature", right? This has been corrected.

Supporting information

Figure S2 caption: "ammonia–carbon dioxide-18O2 ice" can be interpreted that the ice also contains isotopic molecular oxygen. Please replace by "C18O2" or "NH3-C18O2". The same for Figures S3-S6. As a reader, I prefer reading the ices as presented in Table S1.

The captions for Figures S2–S6 have been updated so that the entire molecular formula , e.g., ND_3 or $^{13}CO_2$, is written for all isotopically labelled ices.

Figures S1-S5 are the IR spectrum for freshly prepared ices T=5–10 K. Were these IR spectra recorded as a function of temperature as done for NH3-CO2 ices? What is the purpose of studying the IR spectrum of the isotopically labelled ices? Just to check the red-shift of the absorption bands?

Confirming the red shift resulting from isotopic labeling is a major reason why these data are gathered. This also allows us to verify the isotopic purity of the chemicals delivered to the wafer, particularly important when deuterated amino or hydroxyl groups are used due their tendency to exchange hydrogens with the inner surface of the vacuum tubing and glass capillary array. These spectra also serve as examples of the data used during each experiment to determine the relative abundance of each reactant within the ice, this is the quotative ratio CO_2:NH_3 in Table S1.

Table S6: Which are experiments 1 – 11?

This is a typo, it should read experiments 1–13. The numbered experiments refer to those experiments detailed in Table S1. We have added the following text to avoid confusion: "Because of the orientation of the FTIR and mass spectrometric probes in the experimental chamber, only one may be measured during TPD, these experiments are those for which the PIReToF-MS technique was used."

Minor changes

Throughout the manuscript: Starting a sentence with the number assigned to the carbamic acid (**1**) or ammonium carbamate (**2**) is odd. In those cases, I suggest using the molecular formula. Perhaps the use of the numbers in the text can be reduced.

All incidences of sentences that start with a number have been removed, and small edits have been made throughout the text to reduce usage of the numerical abbreviations when possible.

Page 5, Lines 5 and 34: Ultraviolet (UV) is defined on page 3. It can be written as "…by the intense UV and vacuum UV…" since the acronym VUV does not longer appear in the text. The same for the acronym "UHV" (although is repeated in the SI).

The unused abbreviations VUV and UHV have been removed.

Page 6, Line 35: "carbon dioxide-ammonia-15N and carbon dioxide-18O2- ammonia" can be written as "CO2--15NH3 and C18O2– NH3. The red-shift of 19 cm-1 in spectrum B of Figure S6 is not clearly seen. Maybe if spectrum B is overlapped with A the shift can be perceptible (or not since it is a small shift). Another way of presenting Figure S6 is desired.

Figure S6 has been reformatted to emphasize the position of peaks on the x-axis, and a dashed like has been added with the text:

The dashed vertical line indicates the position (1247 cm^{-1}) of the C–O stretch without isotopic labeling.

To provide this data in quantitative form, the new Table S2 lists the center position of each peak shown in Figure S6.

Page 9, Lines 11 and 24: Parenthesis in NH3 has to be removed: 2 NH3. This has been corrected.

Page 9, Lines 24 and 25: Reactions [5] and [6] can be numbered as [-3] and [-1] since it is the reverse reaction of [3] and [1], respectively.

This has been implemented, and [4] has also been replaced with [−2]. Additionally, the order in which the former reactions [4]–[6] are presented has been changed so that the resulting list of reverse reactions is in the order [−1],[−2],[−3].

Reviewer: 2

Recommendation: Publish in ACS Central Science without change.

Comments: This is a well written and clearly presented paper with an excellent review of previous work. The authors analysis and conclusions are sound. I see no obvious additions required.

We thank the reviewer for their effort and concise review.

Reviewer: 3

Recommendation: Publish in ACS Central Science after minor revisions noted.

Comments:
This manuscript reports on the experimental investigation of the formation and interconversion pathways of carbamic acid and ammonium carbamate along with the dimerization of the former in binary carbon dioxide-ammonia interstellar analog ices. Temperature-resolved condensedphase FTIR spectroscopy was combined with gas-phase detection via isomer-selective singlephoton photoionization time-of-flight mass spectrometry. This study is carried out in experimental conditions that mimic those of the interstellar medium (both in terms of temperature and density) and thus provides an important evidence that these prebiotic molecules can be formed in interstellar ices without energetic radiation. The manuscript is well and clearly written. The conclusions are well sounded and the discussion is well argued. Therefore, in my opinion is deserved to be published.

This study provides an important piece of the origin-of-life puzzle by demonstrating that carbamic acid and ammonium carbamate can be produced in the interstellar medium. I have however a concern about the ice composition. In the interstellar medium, this is expected to be mainly formed by water with other molecules in less abundance. How much does the reaction mechanism rely on the only presence of the two reactants?

This topic was the focus of reference 14, and the following text has been added to the conclusion to inform the reader of this fact and its relevance to real ices:

"Ices observed spectroscopically in molecular clouds are predominantly water unlike model ices employed here; however, the reaction products observed have also been identified with FTIR in model ices composed primarily of water.[14]"

Would the vibrational features be affected by a water-ice environment and how much?

According to reference 14, vibrational features observed with CO_2+NH_3 ice are observed nearly unchanged when ices are composed to about 70% water. Interestingly, irradiation has a larger effect and shifts several of the peaks by a few wavenumbers.
